# Whole farm planning raises profit despite burgeoning climate crisis

**DOI:** 10.1038/s41598-022-20896-z

**Published:** 2022-10-13

**Authors:** Albert Muleke, Matthew Tom Harrison, Rowan Eisner, Peter de Voil, Maria Yanotti, Ke Liu, Xiaogang Yin, Weilu Wang, Marta Monjardino, Jin Zhao, Feng Zhang, Shah Fahad, Yunbo Zhang

**Affiliations:** 1 Tasmanian Institute of Agriculture, University of Tasmania, Newnham Drive, Launceston, TAS 7248 Australia; 2 Queensland Alliance for Agriculture and Food Innovation (QAAFI), The University of Queensland, Gatton Campus, Gatton, QLD 4343 Australia; 3 Tasmanian School of Business and Economics, University of Tasmania, Private Bag 98, Hobart, TAS 7001 Australia; 4 College of Agronomy and Biotechnology, China Agricultural University and Key Laboratory of Farming System, Ministry of Agriculture and Rural Affairs of China, Beijing, 100193 China; 5 Joint International Research Laboratory of Agriculture and Agri-Product Safety, The Ministry of Education of China, Institutes of Agricultural Science and Technology Development, Yangzhou University, Yangzhou, 225009 China; 6 Jiangsu Key Laboratory of Crop Genetics and Physiology, Co-Innovation Centre for Modern Production Technology of Grain Crops, Yangzhou University, Yangzhou, 225009 China; 7 CSIRO Agriculture and Food, Waite Campus, Waite Road, Urrbrae, SA 5064 Australia; 8 College of Resources and Environmental Sciences, China Agricultural University, Beijing, 100193 China; 9 State Key Laboratory of Grassland Agro-Ecosystems, College of Ecology, Lanzhou University, Lanzhou, 730000 China; 10 Hainan Key Laboratory for Sustainable Utilization of Tropical Bioresource, College of Tropical Crops, Hainan University, Haikou, 570228 China; 11 Department of Agronomy, The University of Haripur, Haripur, 22620 Pakistan; 12 Hubei Collaborative Innovation Centre for Grain Industry/Agriculture College, Yangtze University, Jingzhou, China

**Keywords:** Climate-change mitigation, Projection and prediction, Climate change, Climate-change impacts, Environmental health, Governance, Climate sciences, Plant physiology, Drought, Biotic, Plant sciences, Plant stress responses, Heat, Systems biology, Stochastic modelling

## Abstract

The climate crisis challenges farmer livelihoods as increasingly frequent extreme weather events impact the quantum and consistency of crop production. Here, we develop a novel paradigm to raise whole farm profit by optimising manifold variables that drive the profitability of irrigated grain farms. We build then invoke a new decision support tool—*WaterCan Profit*—to optimise crop type and areas that collectively maximise farm profit. We showcase four regions across a climate gradient in the Australian cropping zone. The principles developed can be applied to cropping regions or production systems anywhere in the world. We show that the number of profitable crop types fell from 35 to 10 under future climates, reflecting the interplay between commodity price, yield, crop water requirements and variable costs. Effects of climate change on profit were not related to long-term rainfall, with future climates depressing profit by 11–23% relative to historical climates. Impacts of future climates were closely related to crop type and maturity duration; indeed, many crop types that were traditionally profitable under historical climates were no longer profitable in future. We demonstrate that strategic whole farm planning of crop types and areas can yield significant economic benefits. We suggest that future work on drought adaptation consider genetic selection criteria more diverse than phenology and yield alone. Crop types with (1) higher value per unit grain weight, (2) lower water requirements and (3) higher water-use efficiency are more likely to ensure the sustainability and prosperity of irrigated grain production systems under future climates.

## Introduction

In water-limited environments, irrigated grain crops with adequate nutrition and controlled biotic stresses often yield more grain than rainfed crops on a per unit land area basis, provided irrigation is applied using recommended practices. In Australia, the average annual grain yield of irrigated crops is approximately 4 t/ha compared with 2 t/ha for rainfed crops^1^. Despite this, higher yields of irrigated crops often does not translate into higher profitability, as is the case when water costs are high and grain prices are low. At the farm enterprise level, high input costs—such as would occur when unitary costs of irrigation water are high—and/or low commodity market prices (e.g., grains and fibre) offset or negate positive effects of high yields on profitability. Risk of economic loss in irrigated farming systems is further heightened by (1) longer term climatic change induced primarily by anthropogenic greenhouse gas emissions that (2) contributes to short-term seasonal changes realised by more frequent single or combined weather events and/or altered seasonal distributions of rainfall, such as more rain over summer, and less in winter. Extreme weather events that impact on the profitability and productivity of agricultural systems include droughts, heat waves, extreme cold and extreme rainfall events^2–6^. At the time of writing, south-eastern Queensland in Australia experienced one of the worst flooding crises in history, with damages from the brief but dire rainfall event likely to cost over $AU2.5B in damages^7^.

Cropping systems profitability varies across farm businesses and between years as a function of commodity prices, yields, water use and cost, and variable costs such as repairs, maintenance and labour. Previous studies have demonstrated trade-offs between the potential crop yield, commodity prices, variable costs and water use in determining crop profits^8,9^. For example, high yield per unit area (e.g., cotton bales per hectare) can offset high water-use and variable costs resulting in greater crop profit, whereas high commodity prices (e.g., mungbean prices) can sustain high returns on irrigation investments, despite low crop yield per hectare^10,11^. While commodity prices govern profitability for many rainfed farm businesses^12,13^, water price and use on irrigated farms may have a dominating influence on profitability compared with other variable costs^14^. Climate change and seasonal variability exacerbate the volatility of farm profit both directly (as above) and indirectly by disrupting the irrigation supply/demand ratio, resulting in higher costs of irrigation water during drought periods^15^. For instance, prices for wheat grain and irrigation water in Australia have fluctuated over the past decade from AU$210/t to $435/t^16^ and $20/ML to $550/ML^17^, respectively^10^. (All economic values ($) hereafter are given in Australian dollars (AUD) unless stated otherwise).

Against a background of market and weather volatility, farmers are faced with the need to make tactical decisions (e.g. crop choice and rotation, irrigation scheduling etc.) as well as strategic decisions that influence long term outcomes [e.g. purchasing of machinery, borrowing large sums of money, interventions to improve soil health and carbon, and many others;^4,5,13,18,19^. Such complexity can lead to ‘decision fatigue’; a phenomenon wherein farmers become overburdened with a chronic need to make important but perplexing decisions^20^. To help disentangle and navigate the solution space, various agricultural decision-support systems (DSS) have been developed^20,21^. Amongst other factors, DSS help users better understand the drivers of profitability as a function of variable costs including water use and water price, as well as how the narrowing gap between costs and prices induced by climate change and inflation^22–25^. During drought, irrigation reserves from dams, groundwater bores and natural watercourses may become limiting^26^. This results in increased farm- and regional-level demand for irrigation water^27^, which collectively can exert even further pressure on water reserves, causing water prices to rise. In light of more frequent extreme events such as drought together with the ongoing ‘cost-price squeeze’, farmers must continually adapt just to maintain current profitability, let alone remain prosperous^25^.

Fit-for-purpose decision support systems and advanced digital analytics account for and allow comparisons between climatic, agronomic, financial, social and cultural factors in a simultaneous manner^4,9,28^. Digital tools can help users improve the allocation of available resources (sunlight, water, existing soil nutrients) and inputs (e.g. irrigation water, fertiliser etc.) to improve economic outcomes at the farm scale^9^. Currently however, there are few whole farm decision support tools that facilitate contrasting of tactical (short-term) and strategic (longer-term) economic decisions^20^. In response to this deficit, we built ‘*WaterCan Profit*’, a decision-support tool designed and refined through iterative participative people-centric methods with eight farmer groups spread across the entire Australian Murray-Darling Basin, from South Australia, to northern Victoria and southern Queensland^9^. *WaterCan Profit* includes a mathematical optimiser that allows users to contrast multiple tactical factors, including crop choice, cropping areas, water price, water use, expected grain yields, seasonal climatic conditions, and historical farm management (e.g., crop rotation). *WaterCan Profit* also includes an Investment app that allows strategic analyses through computation of long-term profit (net present value, return on assets, wealth) over the life of the investment^9^. Here, our objectives were to (1) illustrate the capability of the Optimiser app in *WaterCan Profit* through multiple use cases and (2) examine how whole farm profit and optimal crop types and areas change under future climates. We contrast results across a rainfall gradient to gauge how farm business profitability and crop preference may alter across agro-ecological regions under future climates.

## Materials and methods

### Overview

**Figure 1:**
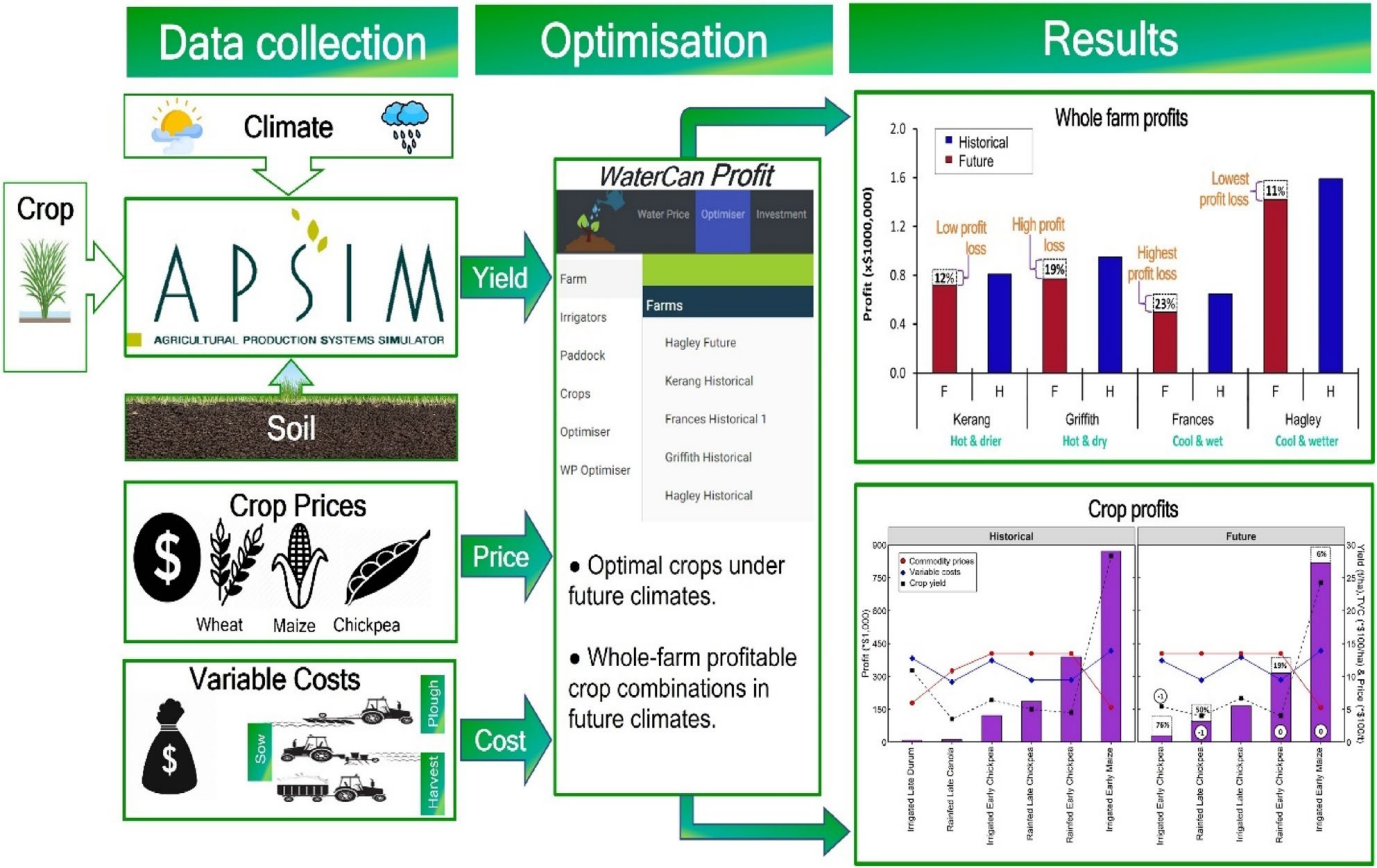
Summary diagram indicating strategic whole farm planning that accounts for crop genetics, biophysical and economic factors can optimise profit despite increasingly frequent extreme climatic events under the climate crisis. Using the Agricultural Production Systems SIMulator (APSIM), we simulated the combined effects of climate change and extreme climatic events on crop yields. A new decision support tool—*WaterCan Profit* optimised crop type and areas that collectively maximised farm profit under historic (1985–2004) and future (2070–2089) climates across four representative irrigated cropping environments in Australia.

We illustrate the capability of *WaterCan Profit* (WCP) in determining profitable whole farm combinations of crops under historic (1985–2004) and future (2070–2089) climates across a rainfall gradient. We selected four representative irrigated cropping environments in Australia, although the conceptual design and systems thinking developed here could be applied anywhere in the world. Biophysical data required as inputs for WCP were obtained (1) using the Agricultural Production Systems SIMulator (APSIM) version 7.10^29,30^ and (2) using data from existing literature on experimental trials and other e.g. ABARES^16^, Poole et al.^31^, GRDC^32^, ABS^33^, DPI^34^. Yields of all crops were simulated with APSIM. Irrigated crops were given water when and as required such that stress associated with water deficit over the crop lifetime was negligible. We conducted this aspect deliberately to ensure that yields of irrigated crops were not limited by water stress. Importantly, simulation of grain yields of individual crops under irrigation management was not the aim of this study; rather, yields were used as inputs to the novel whole farm planning framework we developed and tested, called *WaterCan Profit* (see Fig. 1). While numerous studies have focussed on temporal irrigation management (i.e., scheduling), the purpose of the current study was instead to develop and test a framework for optimally allocating irrigation water over the whole farm and year given expected yields, grain prices, seasonal climate, water costs, variable input costs and all of the other factors influencing profitability at the whole farm scale. Such work is innovative and unique, as (1) *WaterCan Profit* has heretofore not been documented and (2) most studies do not consider production and profit at the whole farm scale.

### Study sites

The Australian grain cropping region occurs across a diverse climatic zone colloquially termed the ‘Wheatbelt’, even though crop types in the region are spatio-temporally diverse and dynamic. Characterised by temperate or Mediterranean climates with winter-dominant rainfall and hot, dry summers, the Wheatbelt spans from south-west Western Australia, across Victoria, southern South Australia, the Midlands of Tasmania, and northwards on the eastern sides of New South Wales and Queensland. Since the 1990s, the Wheatbelt has experienced more frequent heat waves^35–37^ and more frequent spring droughts^38^. We selected four representative irrigation regions to prescribe a climatic gradient, allowing systematic categorisation of climate impacts on profit according to prevailing temperature and precipitation (Table 1).

**Table 1:** Locations and long-term climate characteristics of sites used in this study.

Region	Met. station No	Latitude (°S)	Longitude (°E)	Rainfall (mm)	Minimum temp (°C)	Maximum temp (°C)	Relative climate gradient
Kerang VIC	80,023	− 35.7236	143.9196	387	9.6	22.9	Driest & hottest
Griffith NSW	75,032	− 33.4915	145.5248	398	10.9	24.5	Dry & hot
Frances SA	26,091	− 37.2906	140.8254	584	8	20.2	Wet & cold
Hagley TAS	91,237	− 41.4194	147.1219	680	7.2	18.5	Wettest & coldest

### Climate scenarios

A significant advance of the present study was the approach used to examine the impacts of extreme weather events under future climates. We developed future climate scenarios to account for variability in temperature and rainfall between global climate model (GCM) projections using methods described in Harrison et al.^39^. This approach (1) incorporates mean changes in future climates expected for a region of interest projected by multiple GCMs, (2) accounts for historical climate characteristics for a given site and (3) notwithstanding point (1), generates climate projections with increased variability including more heatwaves, longer droughts and more extreme rainfall events. The study of Harrison et al.^39^, showed that studies which do not explicitly account for the impacts of changes in frequencies of extreme weather events under future climates tend to underestimate the impact of the climate crisis on crop productivity. We suggest that future studies (1) give consideration to changes in the magnitude and frequencies of extreme events within future climate projections and (2) consider how such extremes are accounted for in agricultural systems models.

**Figure 2:**
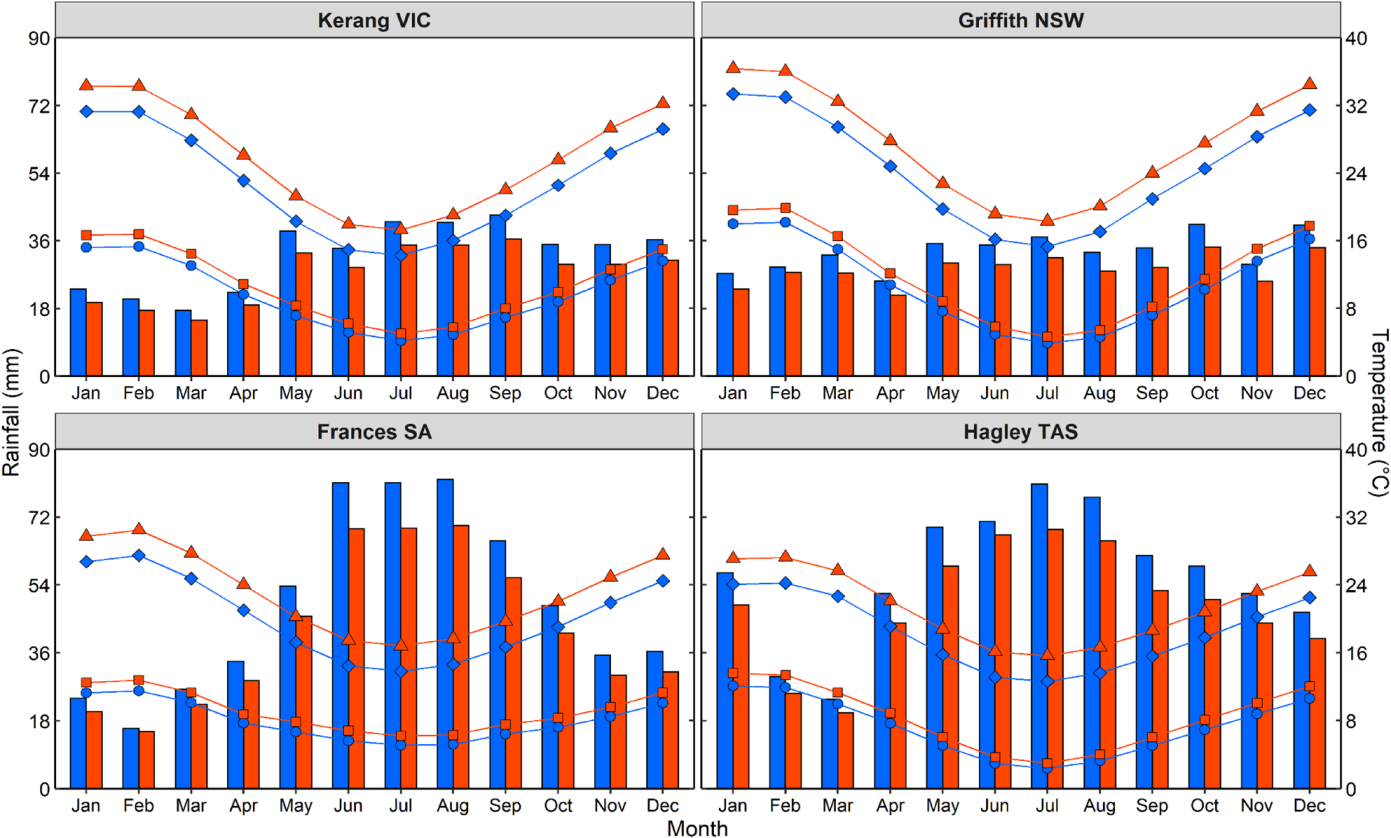
Historical and future climates across four environments in Australian irrigated cropping zones. Averaged across regions, monthly rainfall under future climates (red bars) was reduced by 14% relative to historical rainfall (blue bars); monthly maximum temperature of future climates (red triangles) and monthly minimum temperatures (red squares) were on average 15% greater than corresponding historical daily temperatures (blue points).

We sourced daily data for maximum and minimum temperature, rainfall and solar radiation for the period 1st January 1985 to 31st December 2004 from meteorological archives^40^ and used as historical baselines. All baseline simulations were conducted using an atmospheric CO_2_ concentration of 380 ppm. Future climate scenarios for each site were developed from 1 January 2070 to 31 December 2089 (median time horizon of 2080) using representative concentration pathways 8.5 (RCP8.5)^41,42^, with the numeral representing a radiative forcing of 8.5 W m^−2^ by the end of the century. We adopted RCP8.5 because this scenario most closely aligns with the existing climatic trajectory in Australia^22,27,43^ and more broadly^42^. For each site, historical climate data were used as a basis for modification on a daily time-step to generate future climate data. We adopted “change factors” (CFs) from CCIA^44^ to prescribe monthly average changes in both temperature or rainfall between the historical and future periods, then introduce statistical methods to increase the frequencies of drought, heat waves and extreme rainfall events while preserving monthly average changes in climate. Further detail of approaches used to develop climate scenarios are provided in Harrison et al.^39^. The atmospheric CO_2_ concentration of all future climate scenarios were set at 850 ppm following Collier et al.^45^. Historical and future climate data are summarised in Fig. 2.

### Simulation of crop yield and optimal flowering times

We used the Agricultural Production Systems SIMulator (APSIM) v7.10^29,30^, to simulate the growth and development of durum wheat^46,47^, spring barley^48^, chickpea^49^, canola^50^ and maize^51^. At each location, simulations were repeated with sowing at seven-day intervals, from 1st March to 5th July for winter crops and from 15th September to 19th January for summer crops (Table 2). These simulations were conducted using historical daily climate data from 1 January 1975 to 31 December 2005 and future climate scenario data for the period 1 January 2060 to 31 December 2089, with the first 10 years in each climate scenario discarded to allow for model stabilisation. Crops simulated comprised slow developing (late maturity) and fast developing (early maturity) spring genotypes. Details of genotypic parameters and initialisation settings are shown in Table 2. Soil details for each site shown in Table 3 were adopted from the APSoil database^52^. Plant available soil water at sowing was set to 100% to ensure successful and consistent crop establishment for each sowing date. Irrigated crops received unlimited and timely application of water to negate water deficit stress; nitrogen stress in the model was deactivated to ensure that plant stresses were climatic only. This assumption was made to ensure that (as for irrigation management) yields of irrigated crops were at their potential, unlimited by either abiotic or biotic stresses. This was necessary as to prevent confounding the impact of suboptimal growth of irrigated crops with the expected impact of climate change; thus, the change in yields between current and future climates represented only the change in climatic factors. Optimal flowering periods (OFP) for each site were computed as the flowering dates corresponding to $\ge$ 95% of the maximum 15-day running mean frost-heat yield (FHY), following^53^ and Liu et al.^54^. FHY represents simulated yields accounting for biological impacts of suboptimal (frost) and supraoptimal (heat) stress following Liu et al.^53^, Liu et al.^54^, and Muleke et al.^11^; table reproduced in the supplementary information for clarity (Table S10).

**Table 2:** Crop types, planting densities and genotypes used in this study.

Crop type	Plants (/m^2^)	Genotypes	References
Barley	150	Keel (early), Franklin (late)	Porker et al.^55^, Dreccer et al.^56^, GRDC^57^
Canola	30	Monty (early), Dunkeld (late)	Dreccer et al.^56^, GRDC^57^, Lilley et al.^58^
Chickpea	50	Sonali (early), Dooen (mid-late)	Dreccer et al.^56^, GRDC^57^, Chauhan et al.^59^
Durum wheat	200	Wollaroi (early), Bellaroi (late)	Dreccer et al.^56^, GRDC^57^, Jones^60^
Maize	9	Pioneer_39G12 (early), Pioneer_3237 (late)	Peake et al.^61^, Pembleton et al.^62^

**Table 3:** APSIM soil numbers and soi descriptions for each region.

Region	APSoil No	Soil description
Kerang VIC	1092	Sandy Clay Loam
Griffith NSW	697	Sandy duplex
Frances SA	SE069	Sandy Loam over Brown Clay
Hagley TAS	658	Loam

### Significance statement

We show that strategic whole farm planning that accounts for crop genetics, biophysical and economic factors can enable higher profit despite increasingly frequent extreme climatic events under the climate crisis. We suggest that crop types with (1) higher value per unit grain weight, (2) lower water-use requirements and (3) higher water-use efficiency are more likely to ensure the sustainability and prosperity of irrigated production systems under future climates.

## Demonstrating WaterCan profit: whole farm crop area optimisation to maximise profitability

### WaterCan profit

The decision-support tool WCP comprises three subset apps: a Water Price app, allowing rapid comparison of how crop gross margins vary as a function of water price, an Optimiser app that holistically accounts for expected crop yield, variable costs, water, grain and irrigation price, crop irrigation requirement, rotation, seasonal climatic conditions, and irrigation infrastructure, and an Investment app, allowing insight into time required to payback investments in irrigation machinery, accounting for loan, interest rates, debt, expected life of the machinery, crop rotation, expected yields and variable costs. The three apps were co-designed using a consultative process with farmers and advisors between 2019 and 2022; both initial conceptualisation and refinement of the three apps were conducted with experts from the irrigated grains sector. The maximum number of eight crops for simultaneous comparison in the Optimiser was nominated based on bounded rationality, the idea that human intellectual capacity to rationalise decisions is constrained by the cognitive capacity of the mind^63–65^. A prototype version of WCP is freely available online (www.watercanprofit.com.au); on first application, users should create a username and password that can be subsequently used to login to the decision-support tool.

### Screening genotype by management options for use in the Optimiser

**Figure 3:**
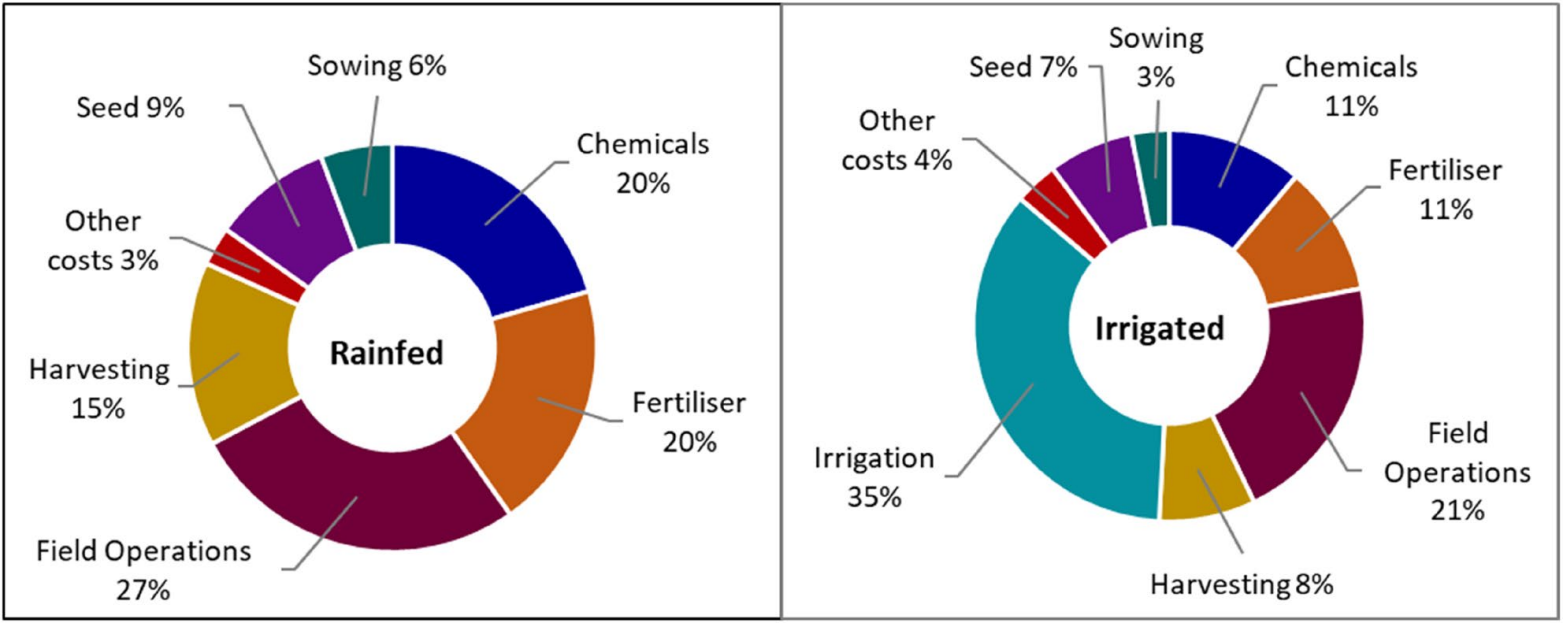
Average distribution of farm business variable costs for early and late maturity genotypes of durum wheat, barley, canola, chickpea and maize under irrigated and dryland conditions across four representative regions in the Australian irrigated cropping zones.

A total of 16 genotype x management options were chosen for each site, including a range of crop types, genotypes and watering regime. The options comprised rainfed genotypes for barley, irrigated genotypes for maize and, rainfed and irrigated genotypes for canola, durum wheat and chickpea (Table S8). To prioritise profitable genotype x environment by management options across sites for use in the Optimiser, we computed gross margins (GMs) using Eq. (1), following Malcolm et al.^66^:

1 $$GM \,per\, unit\, area \left( {\$ /ha} \right) = \left[ {grain \,yield \,\left( {t/ha} \right) \times grain\, price \,\left( {\$ /t} \right)} \right]{-} TVC \left( {\$ /ha} \right)$$

where TVC represents total variable cost and includes outgoing payments associated with sowing, seed, fertiliser, chemicals (herbicides and fungicides), field operations (i.e., cultivation, spraying, casual labour, fuel and repairs), irrigation water use, casual labour, harvesting (i.e., stripping, windrowing, packaging and freight) and other selling expenses (i.e., levies). Variable costs were sourced from ABARES^16^, GRDC^67^, McKellar et al.^8^, Harrison et al.^9^, Ash et al.^68^, NRE^69^, PIRSA^70^ and^71^. Water costs were derived from ABS^17^, BoM^72^ and^73^. Irrigation water use constituted the highest proportion of TVC for the irrigated crops, whereas for the rainfed crops, fertilisers, chemicals and field operations dominated farm cost profiles (Fig. 3).

Commodity prices were estimated from ABARES^16^, GRDC^74^ and ABS^33^, ABS^75^ for the period 1992–2021. Historical nominal prices ($/t) were adjusted for inflation using the consumer price index (CPI) computation shown in Eq. (2). Inflation adjusted (real) prices across sites and climates are shown in Table 4.

2 $$Real\, price = \left\{ {\left( {\frac{{CPI_{current \,year} - CPI_{base \,year} }}{{CPI_{base \,year} }}} \right) \times NP_{base \,year} } \right\} + NP_{base year}$$

where: CPI = consumer price index for the baseline and current years ^75^. NP = Nominal prices not adjusted for inflation.

**Table 4:** Real prices ($/t) for crops across a range of representative environments in Australian irrigated cropping regions.

Crop	Low	Median	High
Chickpea	441	790	1348
Canola	560	708	1086
Wheat	332	448	596
Maize	273	418	528
Barley	247	434	472

Price ranges were specified from 1992 to 2022 using data sourced from ABARES^16^ and ABS^33^.

Crops with the highest GMs are shown in Table S5 and were selected for further analysis using the Optimiser in WCP. Real prices (Table 4) and variable costs (Fig. 3) were deliberately held constant for historical and future climates to avoid confounding the effects of climate change with effects of changes in prices.

### Whole farm optimisation of crop areas to maximise profit using the Optimiser

To examine profitability on an area ($/ha) basis for the selected crops, we used the WCP Optimiser Apps for determining the most profitable farm systems. The Optimiser requires specification of the following information:

- *Farm*: farm name, geographical locations and rainfall (winter and summer—Table S9).
- *Irrigation*: annual allocation of irrigation water (Table S6) and water prices (Table S7).
- *Paddock*: for simplicity we modelled one paddock as the whole farm and allowed the Optimiser to compute the optimal areas. For ease of interpretation of results, we assume a constant farm size of 100 ha in all cases.
- *Crops*: details for the selected crops, growing season (winter and summer), watering regime (rainfed and irrigated) and irrigation water application rates are shown in Table S7, while crop yields at OFP generated from APSIM, price and TVC are shown in Tables 6 and S5.
- *Optimiser*: optimisation based on $/ha conducted for each site and climate scenario.

## Results

### Grain yields at optimal flowering periods (OFPs) under historical climates

Across sites, sowing periods and years, the irrigated late maturity (hereafter, “late”) winter and early maturity (hereafter, “early”) summer crop genotypes had the highest mean yields, while the dryland early winter and late summer genotypes had the lowest peak mean yield (Tables 5, S1, S2, S3 and S4). The highest peak mean yields occurred at the high rainfall site of Hagley in the State of Tasmania by the irrigated early maturity summer maize crop and most winter crops (spring barley, durum wheat, chickpea and canola). The lowest peak yields occurred at the lower rainfall sites of Griffith (New South Wales) for the dryland early maturity winter genotypes (canola, barley and chickpea) and at Kerang (Victoria) by the dryland late summer genotypes of maize and the dryland early maturity winter genotypes of durum wheat. The length of the optimal flowering window varied between crop genotypes under irrigation and rainfed conditions across sites (Tables 5, S1, S2, S3 and S4). Generally, irrigation extended the OFP window compared with dryland crops, particularly for the late winter genotypes, consistent with findings by Muleke et al.^11^. The latest OFP was at Hagley for irrigated late winter genotypes of canola (25 September), chickpea (4 October), spring barley (5 November) and durum wheat (12 November).

**Table 5:** Yield, optimal flowering periods and average water applied per annum of irrigated and dryland durum wheat across a range of representative environments in Australian irrigated cropping regions under historical (H = 1985–2004) and future (F = 2070–2089) climates.

Region	Genotype	Regime	Period	Optimal range of sowing dates		Maximum Yield	Optimal range of flowering period		Crop Duration	Average irrigation per year
				Earliest	Latest	(kg/ha)	Start	Close	(Days)	(ML)
Kerang	Early	Dryland	H	10-May	24-May	3521	15-Sep	29-Sep	128	–
			F	17-May	17-May	2174	28-Aug	31-Aug	103	–
		Irrigated	H	7-Jun	5-Jul	6254	11-Oct	1-Nov	126	894
			F	7-Jun	21-Jun	4536	5-Oct	10-Oct	120	845
	Late	Dryland	H	26-Apr	10-May	4019	12-Sep	22-Sep	139	–
			F	26-Apr	10-May	2584	29-Aug	2-Sep	125	–
		Irrigated	H	17-May	28-Jun	6800	12-Oct	28-Oct	148	1025
			F	10-May	7-Jun	5004	10-Oct	15-Oct	153	983
Griffith	Early	Dryland	H	10-May	24-May	3630	15-Sep	17-Sep	128	–
			F	10-May	17-May	2169	28-Aug	3-Sep	110	–
		Irrigated	H	24-May	5-Jul	6309	4-Oct	15-Oct	133	1233
			F	7-Jun	21-Jun	4622	5-Oct	6-Oct	120	1138
	Late	Dryland	H	3-May	10-May	3856	20-Sep	30-Sep	140	–
			F	3-May	3-May	2460	30-Aug	5-Sep	119	–
		Irrigated	H	17-May	28-Jun	6906	14-Oct	23-Oct	150	1400
			F	17-May	14-Jun	5182	10-Oct	11-Oct	146	1321
Frances	Early	Dryland	H	14-Jun	28-Jun	4207	19-Oct	26-Oct	127	–
			F	21-Jun	5-Jul	2924	30-Sep	4-Oct	101	–
		Irrigated	H	14-Jun	5-Jul	5252	27-Oct	2-Nov	135	532
			F	21-Jun	5-Jul	4312	24-Oct	23-Oct	125	486
	Late	Dryland	H	24-May	7-Jun	4748	24-Oct	2-Nov	153	–
			F	24-May	14-Jun	3515	1-Oct	7-Oct	130	–
		Irrigated	H	31-May	5-Jul	5798	6-Nov	9-Nov	159	615
			F	7-Jun	5-Jul	4605	29-Oct	25-Oct	144	575
Hagley	Early	Dryland	H	14-Jun	5-Jul	5479	30-Oct	8-Nov	138	–
			F	21-Jun	5-Jul	3934	18-Oct	28-Oct	119	–
		Irrigated	H	28-Jun	5-Jul	7956	3-Nov	10-Nov	128	884
			F	28-Jun	5-Jul	6494	29-Oct	2-Nov	123	853
	Late	Dryland	H	17-May	28-Jun	5981	2-Nov	11-Nov	169	–
			F	10-May	21-Jun	4194	20-Oct	31-Oct	163	–
		Irrigated	H	7-Jun	5-Jul	8718	12-Nov	15-Nov	158	1017
			F	14-Jun	5-Jul	6847	4-Nov	5-Nov	143	1013

### Yield and optimal flowering periods under future climates

**Figure 4:**
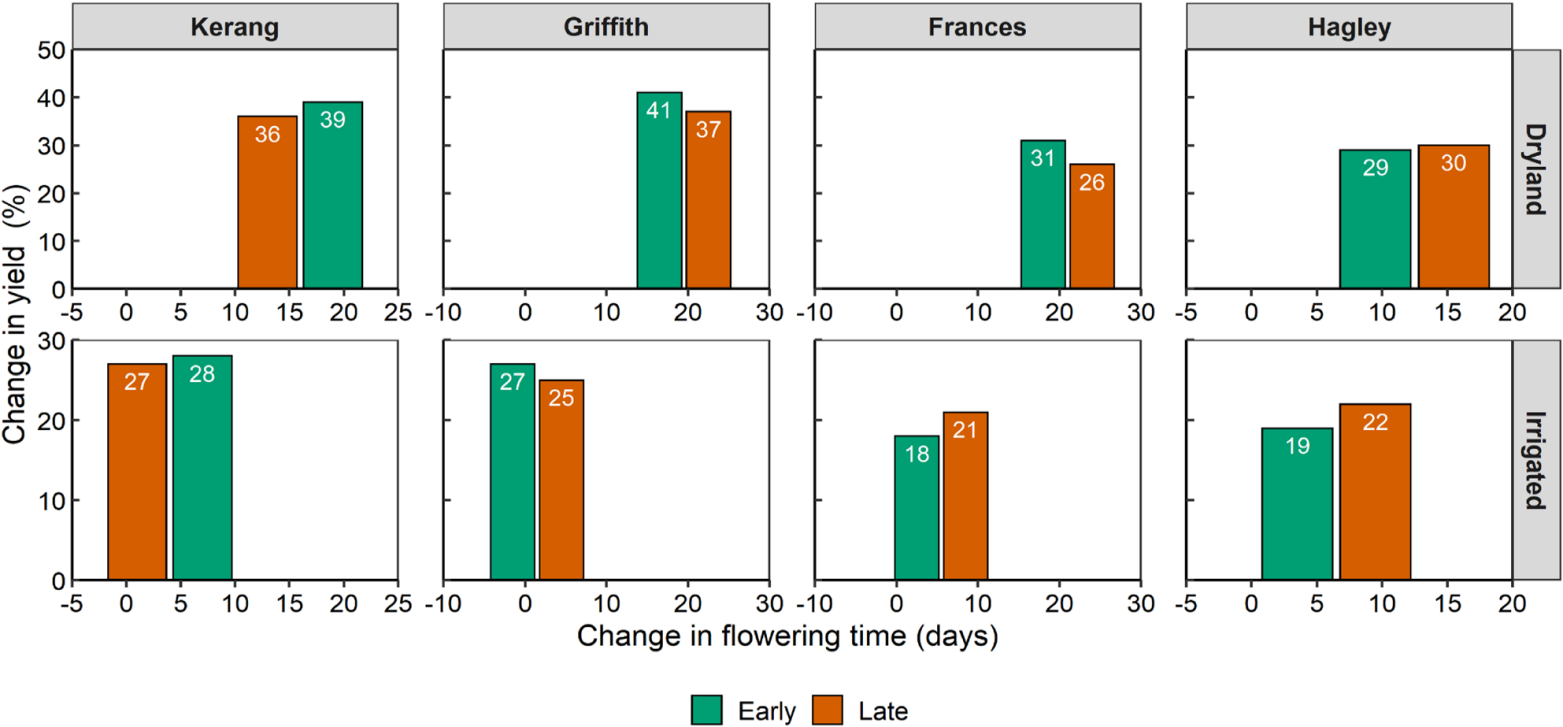
Impacts of future climates on optimal flowering times and yield. Columns show relationships between shift in optimal flowering duration (days) and percentage reduction in peak yield for early (green columns) and late (brown columns) maturity genotypes of durum wheat in dryland (top row) and irrigated (bottom row) conditions across a range of representative environments in Australian irrigated cropping regions. Future (2070–2089) climates truncated crop lifecycles, shifting forward flowering times relative to historical (1985–2004) climates. Irrigation partially offset the forward shifts in flowering by lengthening lifecycles, resulting in later flowering periods in some regions (indicated by negative values). Regions are depicted along a rainfall gradient, from the lowest average annual rainfall (Kerang, 387 mm) to the highest (Hagley, 680 mm).

In general, future climates reduced grain yield and shifted forwards OFPs, primarily due to increasing temperature. Forward shifts in OFPs (Figs. 4, S1, S2, S3 and S4) were greater for dryland scenarios (6–39 days) compared with those under irrigation (− 35–16 days) across crops and sites. For most regions, the decline in yields at the OFP (i.e., peak yields) were greater under dryland conditions (− 23%) compared with that under irrigation (− 16%). The largest yield losses occurred at the hot dry regions of Griffith (41%; Fig. 4) and Kerang (39%) because these regions had shorter crop durations under future climates (92 and 89 days from start of sowing to start of flowering averaged across crops; Tables 5, S1, S2, S3 and S4). On average, peak yield declined across all sites and management regimes by 21% under the future climate. These results collectively indicate that indirect effects of irrigation via crop duration alleviates detrimental climatic impacts on yield, in addition to direct effects of irrigation relieving crop water stress per se. These indirect effects were emergent properties from APSIM (not APSIM inputs); the model deterministically accounts for daily temperature and thermal sum on crop lifecycle. APSIM is specifically designed to account for climatic influences on crop development and is thus ideal for the purpose of the present study. Changes in optimal flowering windows are shown in Table 5 and were simulated with APSIM using a range of input years for the historical (1985–2004) and future (2070–89) climate horizons. In general, larger shifts in OFP resulted in larger declines in yield, though this relationship were more obvious for the dryland scenarios.

### Screening of crops by gross margins

**Figure 5:**
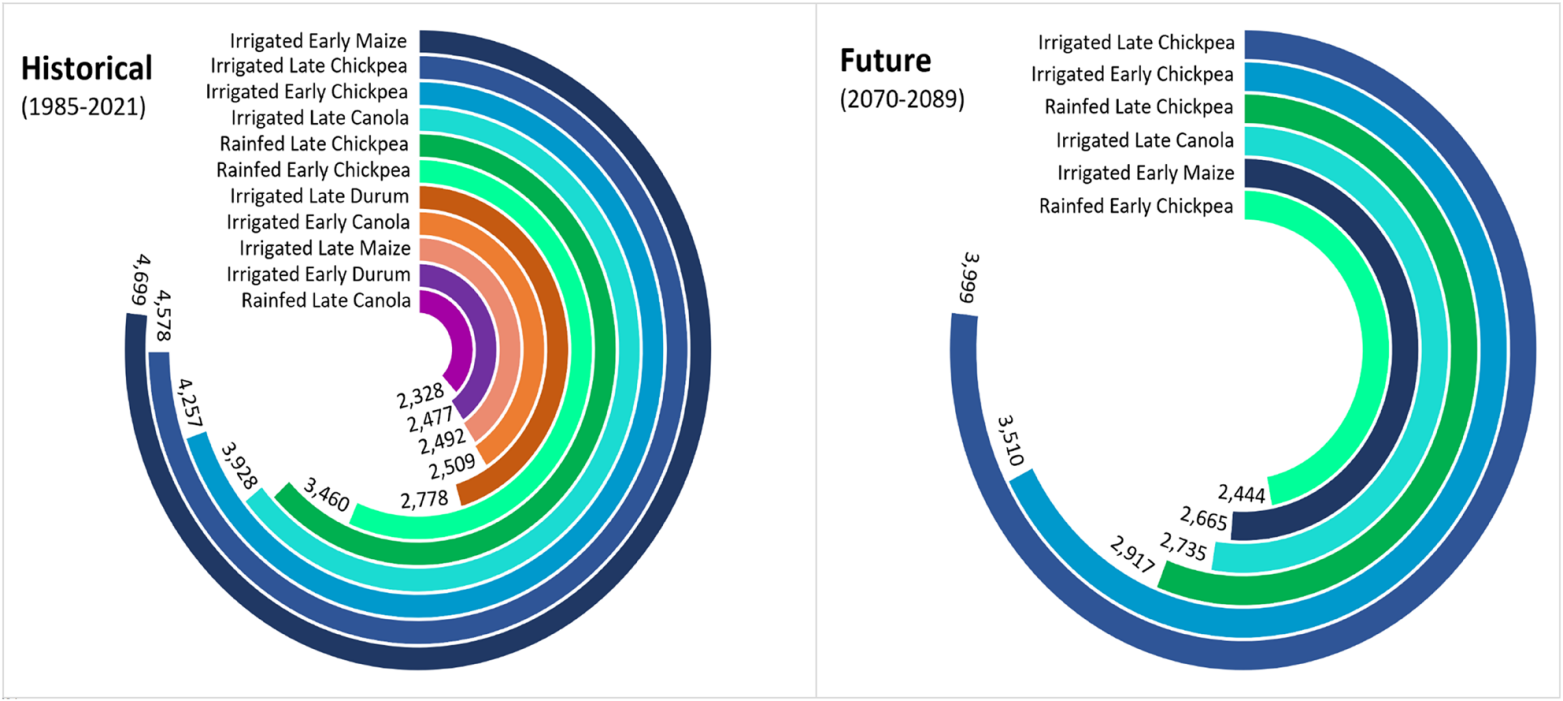
Most profitable farm systems under historical (1985–2021) and future (2070–2089) climates for early and late crop genotypes in dryland and irrigated conditions in a hot dry Australian cropping region with annual rainfall < 400 mm (Kerang, Victoria). The numerical values represent gross margins. Crops that were not profitable (negative gross margin) under future climates were removed. Other sites are shown in Table S5.

**Figure 6:**
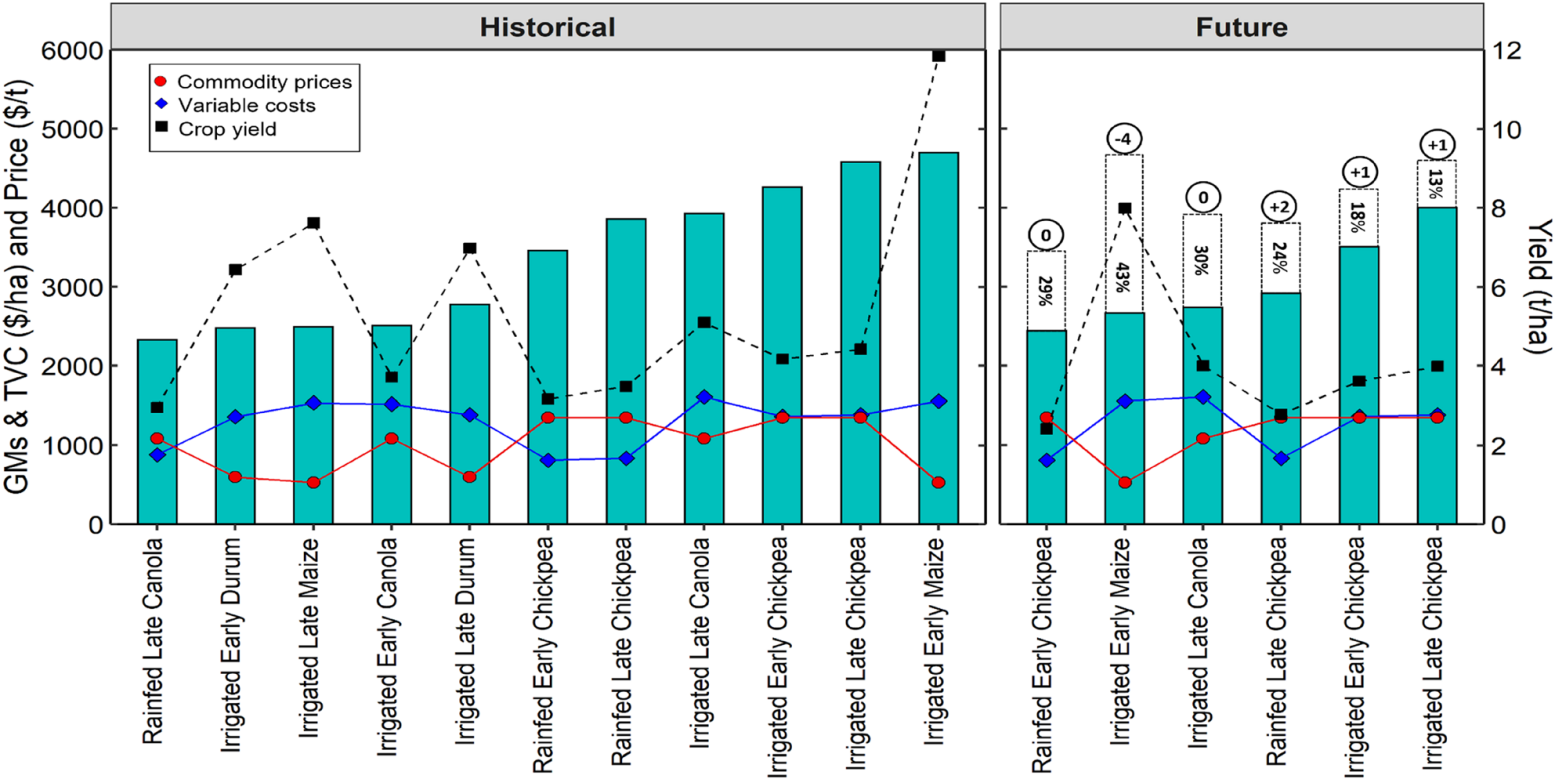
Gross margins as a function of grain prices, yield and variable costs for a hot dry region (Kerang) under historical and future climates. Green bars represent gross margins (GMs) for the most profitable farm systems. Black dotted line represents peak grain yield at optimal flowering time. The red line represents long-term high grain prices, while the blue line represents the highest total variable costs (TVC). Dotted bar segments represent percentage reduction in GMs and numbers in black circles represent change in crop rank relative to historical climates (positive and negative denote shift to the right and left, respectively).

In general, future climates reduced the number of profitable farm systems by 3–5 across regions (Figs. 5, 6, Tables 6, and S5). Crop gross margins declined by − 21% under future climates (2070–2089) across regions and water regimes (Tables 6 and S5). The cool temperate regions (e.g., Hagley in Tasmania) had the highest number of profitable farm systems (13 and 10 under historical and future climates respectively), attributed to the high peak mean yields achieved by majority of the crops at this site. Other regions had up to 11 farm systems under historical climates and 6 farm systems for the future scenarios (Tables 6 and S5). Chickpea was often the most profitable crop when assessed across the regions and climates, mainly due to relatively high commodity price compared with other crops (Tables 4, 6 and S5). Similar trade-offs between grain prices, peak yield and variable costs in determining the highest GMs were evident for all regions. For example, at Kerang under historical climates (Fig. 6 and Table 6), effects of higher TVC for irrigated early maize ($1557/ha) on GM were partially negated by higher yield (11.8 t/ha), resulting in greater GM ($4,699/ha). In contrast, higher grain prices for irrigated late chickpea ($1348/t) resulted in relatively high GMs ($4578/ha), even though chickpeas predominantly had lower yields than irrigated maize. Overall, the average decline in crop gross margins was greater for dryland scenarios compared with irrigated conditions under future climates; GM reduction ranged from 11 to 24% in dryland environments and from 4 to 14% under irrigation (Tables 4, 6 and S5).

**Table 6:** Crop gross margin ranking under current and future climates as a function of yields, real prices and variable costs for a hot dry region (Kerang) for historical (white rows, 1985–2021) and future (itallic rows, 2070–2089) climates (other regions shown in Table S5).

Rank	Crop	Genotype	Regime	Yield (t/ha)			Net sale price ($/t)			Total variable cost ($/ha)			Gross Margins ($/ha)		
				Low	Median	High	Low	Median	High	Low	Median	High	Low	Median	High
1	Maize	Early	Irrigated	3.6	5.9	11.8	273	418	528	893	1290	1557	78	1182	4699
2	Chickpea	Late	Irrigated	1.3	2.2	4.4	441	790	1348	488	765	1385	97	982	4578
3	Chickpea	Early	Irrigated	1.3	2.1	4.2	441	790	1348	485	758	1365	67	888	4257
4	Canola	Late	Irrigated	1.7	2.7	5.1	560	708	1086	588	1041	1610	359	846	3928
5	Chickpea	Late	Rainfed	1.0	1.7	3.5	441	790	1348	428	552	835	32	823	3856
6	Chickpea	Early	Rainfed	1.0	1.6	3.2	441	790	1348	422	543	810	− 3	708	3460
7	Durum	Late	Irrigated	2.2	3.6	7.0	332	448	596	446	833	1384	290	771	2778
8	Canola	Early	Irrigated	1.3	2.0	3.7	560	708	1086	568	1004	1518	146	391	2509
9	Maize	Late	Irrigated	2.3	3.8	7.6	273	418	528	890	1277	1533	−265	314	2492
10	Durum	Early	Irrigated	2.1	3.3	6.4	332	448	596	440	821	1360	242	660	2477
11	Canola	Late	Rainfed	0.9	1.5	3.0	560	708	1086	451	686	879	46	360	2328
*1*	*Chickpea*	*Late*	*Irrigated*	*1.2*	*2.0*	*4.0*	*441*	*790*	*1348*	*488*	*765*	*1385*	*40*	*812*	*3999*
*2*	*Chickpea*	*Early*	*Irrigated*	*1.1*	*1.8*	*3.6*	*441*	*790*	*1348*	*485*	*758*	*1365*	− *7*	*670*	*3510*
*3*	*Chickpea*	*Late*	*Rainfed*	*0.8*	*1.4*	*2.8*	*441*	*790*	*1348*	*428*	*552*	*835*	− *60*	*548*	*2917*
*4*	*Canola*	*Late*	*Irrigated*	*1.2*	*2.0*	*4.0*	*560*	*708*	*1086*	*588*	*1041*	*1610*	*85*	*376*	*2735*
*5*	*Maize*	*Early*	*Irrigated*	*2.4*	*4.0*	*8.0*	*273*	*418*	*528*	*893*	*1290*	*1557*	− *238*	*378*	*2665*
*6*	*Chickpea*	*Early*	*Rainfed*	*0.7*	*1.2*	*2.4*	*441*	*790*	*1348*	*422*	*543*	*810*	− *103*	*411*	*2444*

## Optimisation of crop type and area across the farm to maximise profitability

### Historical climates

**Figure 7:**
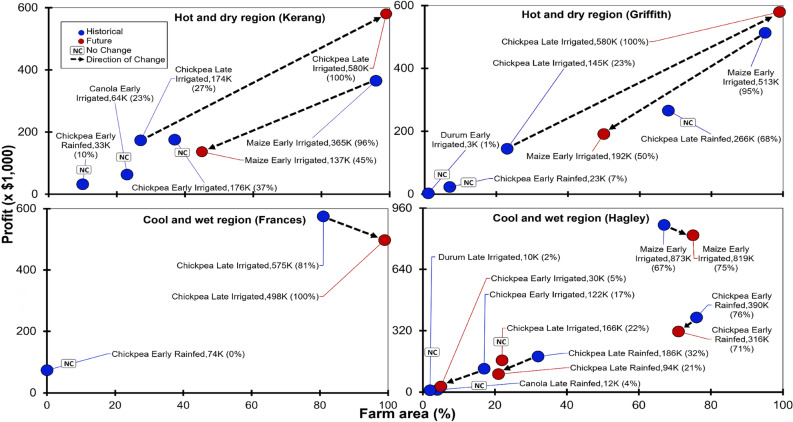
Change in farm profit and relative portion of whole farm area caused by climate change across irrigated grains regions of Australia under historical and future climates. Blue points indicate values for historical climates, red points denote future climates and arrows show shifts caused by climatic change. NC = no change between historical and future climates.

**Figure 8:**
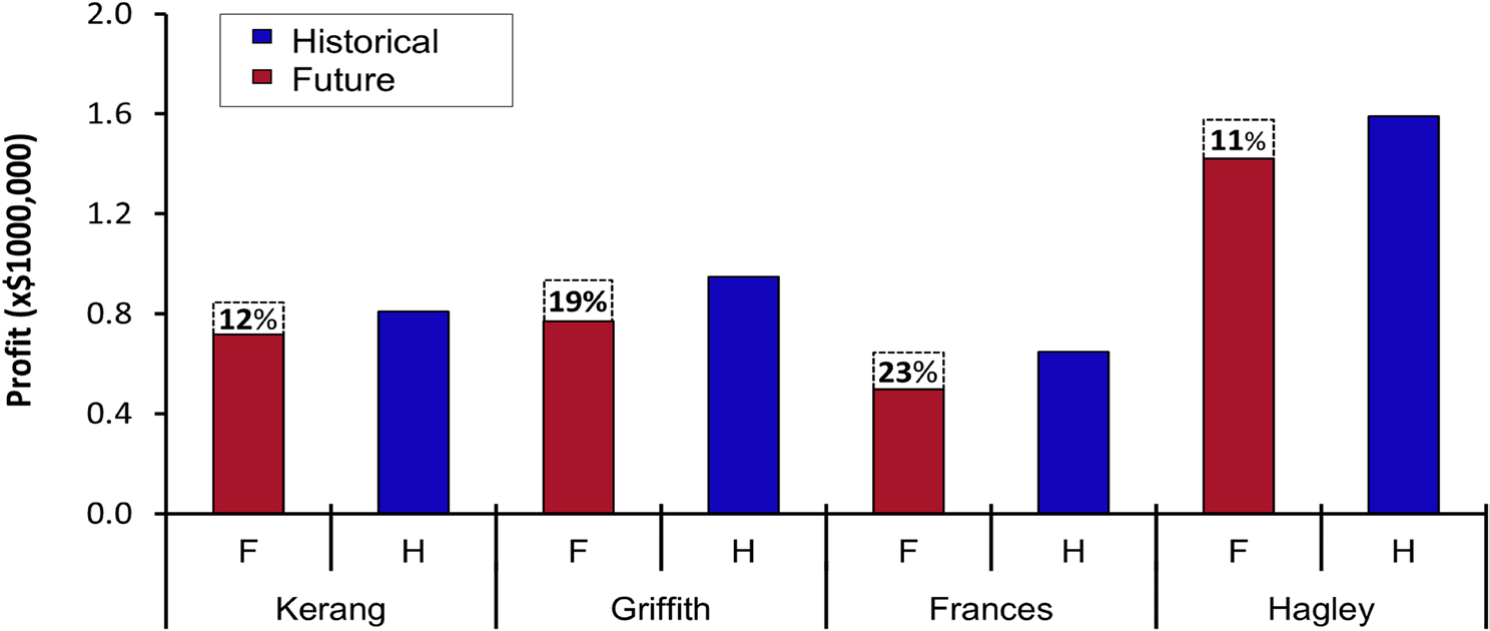
Average whole farm profitability across a rainfall gradient for four representative environments in Australian irrigated cropping regions under historical (H = blue bars) and future (F = red bars) climates. Dotted bar segments represent percentage reduction in average whole farm profit.

In general, higher crop prices generated the most profitable outcomes, often resulting in fewer crops across regions and climates (Table 7). The cool temperate region of Hagley attained the highest whole farm profit ($1.6 M) from five winter crops and summer maize (Figs. 7, 8 and Table 7). The relatively high rainfall mild temperature region of Frances in South Australia had the lowest profit at $0.65 M (40% relative to cool temperate region) due to a combination of rainfed early and irrigated late chickpea (Figs. 7, 8 and Table 7). Compared with drier and hotter regions around Griffith ($0.9 M; 56%) and Kerang ($0.8 M; 50%), low profitability in the Frances region was in part due to high irrigation water costs which meant that except for chickpeas, all crops were not economically viable (Table S7).

**Table 7:** Farming systems and crop planting areas that maximise whole farm profit under historical and future climates.

Region	Period	Crop	Price ($/t)	Yield (t/ha)	TVC ($/ha)	Water Applied			Water Cost ($/ha)	Gross Margin		Area (%)	Profit ($)	Profit (%)
						(ML/ha)	(mm)	(%)		($/ha)	($/ML)			
Kerang	Historical	Maize Early Irrigated	528	11	1557	5	500	56	475	3801	760	96	364,910	45
		Chickpea Early Irrigated	1348	4.8	1365	4	400	17	380	4743	1186	37	175,489	21
		Chickpea Late Irrigated	1348	6.1	1385	5	500	16	475	6425	1285	27	173,482	22
		Canola Early Irrigated	1086	4.3	1518	4	400	11	380	2761	690	23	63,498	4
		Chickpea Early Rainfed	1348	3	835	0	0	0	0	3209	0	10	32,090	8
		**Total**				855 (ML)							809,469	
	Future	Chickpea Late Irrigated	1348	5.7	1385	5	500	58	475	5800	1160	100	579,966	81
		Maize Early Irrigated	528	10.1	1557	8	800	42	760	3038	380	45	136,729	19
		**Total**				860 (ML)							716,696	
Griffith	Historical	Maize Early Irrigated	528	14.7	1567	10	1000	87	800	5400	540	95	512,954	54
		Chickpea Late Rainfed	1348	3.5	815	0	0	0	0	3903	0	68	265,404	28
		Chickpea Late Irrigated	1348	6	1384	6	600	13	480	6279	1047	23	144,426	15
		Chickpea Early Rainfed	1348	3	799	0			0	3245	0	7	22,715	2
		Durum Early Irrigated	596	7.6	1360	5	500	0	400	2755	551	1	2755	0.3
		**Total**				1093 (ML)							948,254	
	Future	Chickpea Late Irrigated	1348	5.7	1384	6	600	55	480	5794	966	100	579,355	75
		Maize Early Irrigated	528	11.7	1567	10	1000	45	800	3825	382	50	191,250	25
		**Total**				1100 (ML)							770,605	
Frances	Historical	Chickpea Late Irrigated	1348	6.4	1231	3	300	100	330	7097	2366	81	574,854	89
		Chickpea Early Rainfed	1348	3.5	835	0	0	0	0	3883	0	0	73,777	11
		**Total**				243 (ML)							648,631	
	Future	Chickpea Late Irrigated	1348	4.8	1231	3 (100%)	300	100	330	4973	1658	100	497,337	100
		**Total**				860 (ML)							497337	
Hagley	Historical	Maize Early Irrigated	528	28.3	1390	8	800	87	520	13,016	1627	67	872,089	55
		Chickpea Early Rainfed	1348	4.5	947	0	0	0	0	5119	0	76	389,044	24
		Chickpea Late Rainfed	1348	5	944	0	0	0	0	5796	0	32	185,472	12
		Chickpea Early Irrigated	1348	6.4	1244	4	400	11	260	7160	1790	17	121,712	8
		Canola Late Rainfed	1086	3.5	917	0	0	0	0	2884	0	4	11,536	0.7
		Durum Late Irrigated	596	10.9	1275	5	5	2	325	4905	981	2	9811	0.6
		**Total**				614 (ML)							1,589,664	
	Future	Maize Early Irrigated	528	24.3	1390	8	800	85	520	10,910	1364	75	818,288	58
		Chickpea Early Rainfed	1348	4	947	0	0	0	0	4445	0	71	315,595	22
		Chickpea Late Irrigated	1348	6.7	1294	4	400	13	260	7508	1877	22	165,166	12
		Chickpea Late Rainfed	1348	4	944	0	0	0	0	4448	0	21	93,408	7
		Chickpea Early Irrigated	1348	5.4	1244	3	300	2	195	5800	1933	5	29,001	2
		**Total**				703 (ML)							1,421,458	

Results are shown for a range of representative environments in Australian irrigated cropping regions under historical (H = 1985–2021) and future (F = 2070–2089) climatic conditions.

TVC = total variable cost, percentage column under water applied denote annual water allocation used for that crop to attain peak yields. Regions are depicted along a rainfall gradient, from the lowest average annual rainfall (Kerang, 387 mm) to the highest (Hagley, 680 mm).

**Figure 9:**
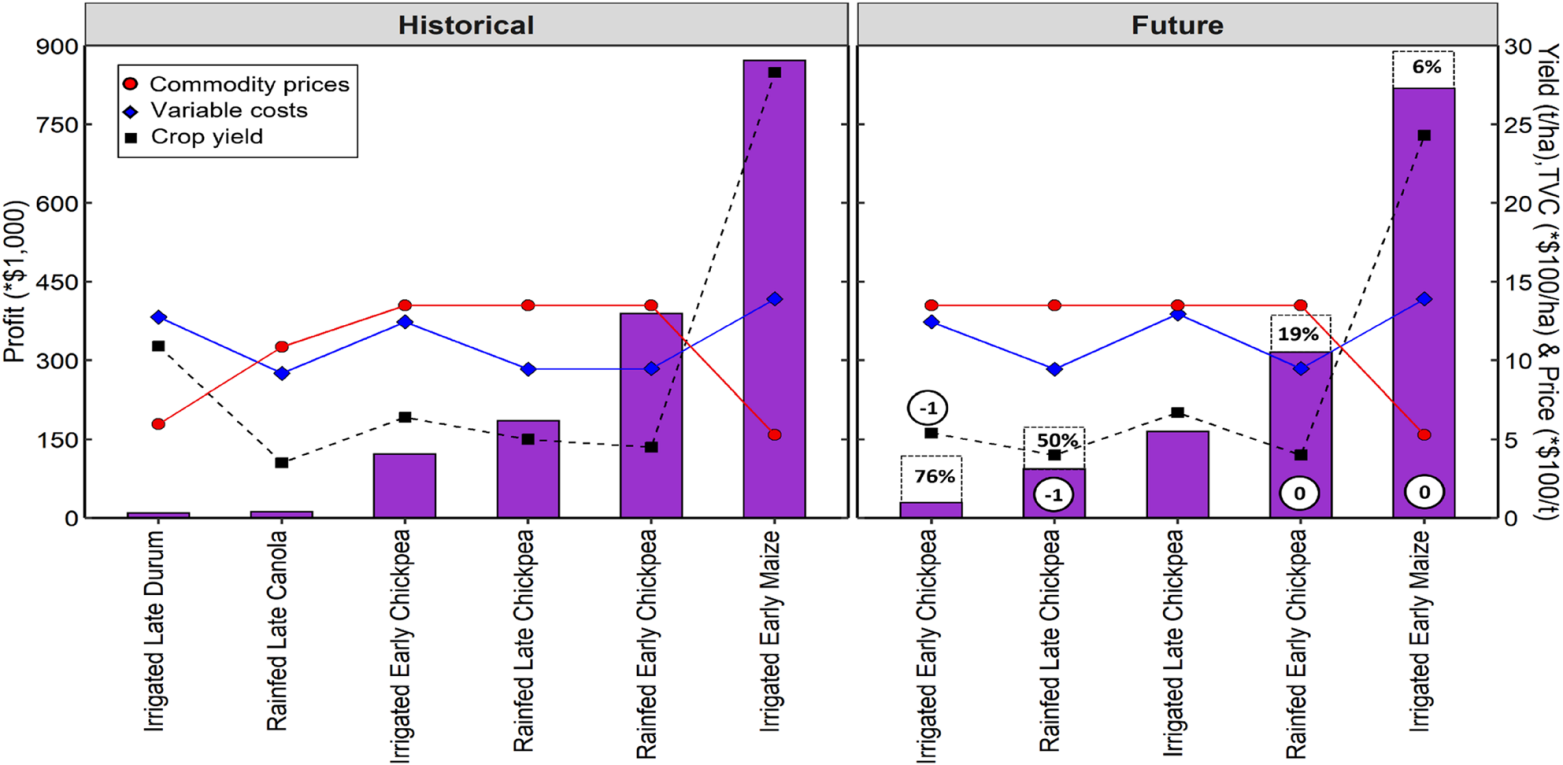
Farm systems profitability as a function of grain price, yield and variable cost for a cool wet region under historical and future climates. Purple bars represent profit of the most profitable farm systems. Black dotted lines represent peak grain yield attained during the optimal flowering window. The red line represents long-term high grain prices, while the blue line represents the highest total variable costs (TVC). Dotted bars represent percentage reduction in profit and numbers in black circles represent change in crop profitability ranking relative to the historical climates.

**Figure 10:**
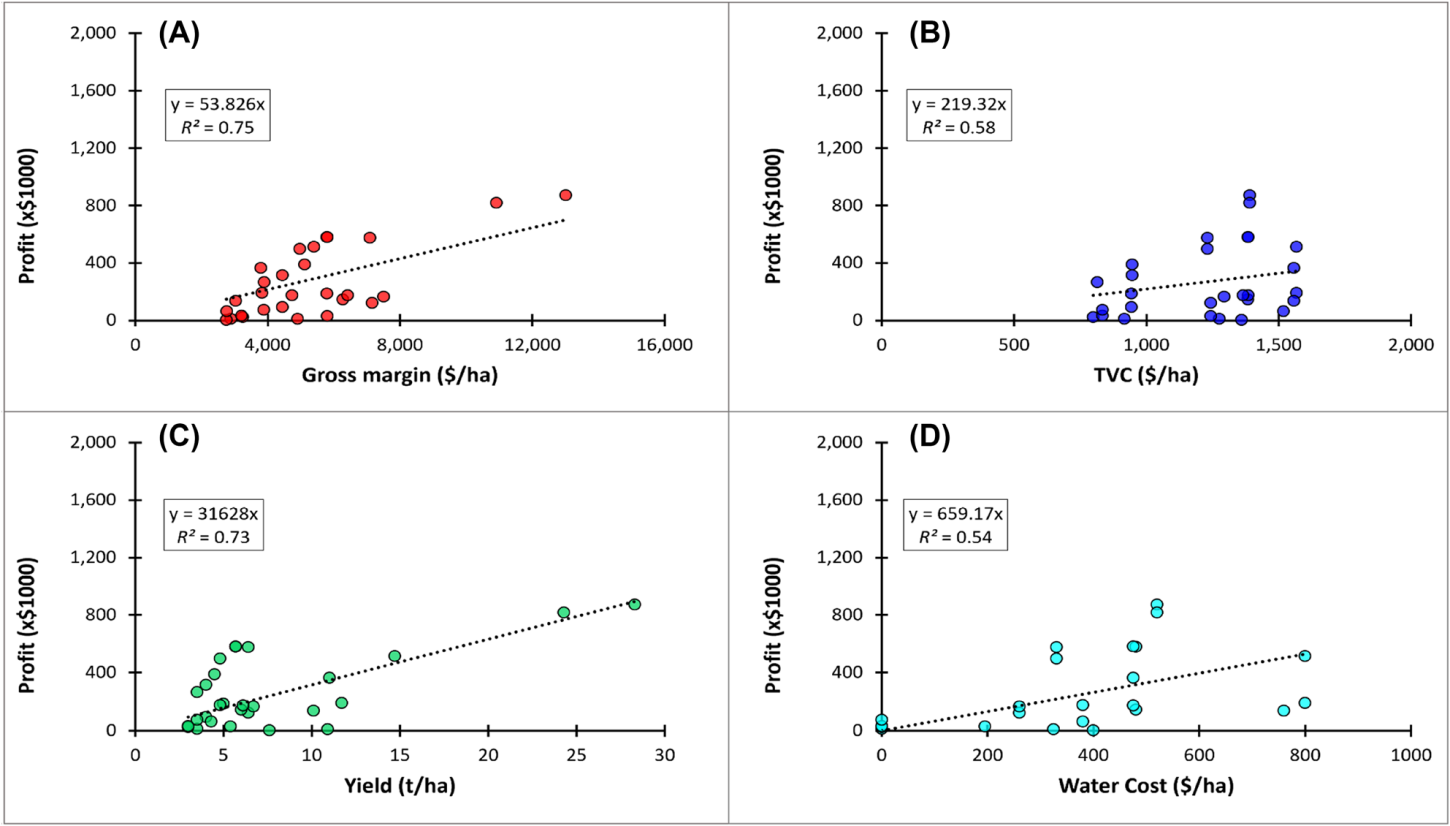
Relationships between profit and gross margin (**A**), total variable costs (**B**), yield (**C**) and water costs (**D**) at high prices for the most profitable whole farm systems across representative environments in irrigated grain regions of Australia under historical and future climates.

Irrigated early maize was the most profitable crop at Hagley ($0.87 M, 55% of whole farm profit; Fig. 9 and Table 7), Griffith ($0.5 M; 54%) and Kerang ($0.36 M; 45%), whereas winter sown irrigated late chickpea was most profitable at Frances ($0.57 M; constituting 89% of farm returns). Overall, irrigated late chickpea was often the most profitable crop across regions (Tables 7). In general, whole farm profitability was most strongly associated with gross margin (*R*^2^ = 0.75) and yield (*R*^2^ = 0.73) compared with TVC (*R*^*2*^ = 0.58) and water cost (*R*^2^ = 0.54), as illustrated in Fig. 10.

### Impact of future climates on extent of profitable farm systems

Across regions, future climates reduced the number of profitable farm systems by 1–3 and profitability by − 16% (Figs. 7, 8 and Table 7). The highest decline in the number of profitable farm systems (i.e., three systems no longer profitable under future climates) was at the dry and hot regions of Griffith and Kerang, whereas the lowest reduction in the number of profitable cropping systems (1 system) was in the relatively wet and cold regions. Chickpeas and irrigated early maize were the most climate-resilient profitable crops across the regions, owing to higher potential yield for irrigated early maize and higher prices with relatively low water requirement for chickpeas. Interplay between commodity price, yield and variable costs observed for all regions reduced the total number of most profitable crop options from 35 to 10 (Fig. 9 and Table 7). For example, in the Hagley region (Fig. 9 and Table 7), relatively higher yield of irrigated early maize offset the high TVC and low grain price of this crop, resulting in greater profits.

### Change in whole farm profit under future climates

In general, future climates induced substantial changes in profits and area sown for profitable farm systems at the drier regions (by − 63 to + 301% and − 51 to + 77% respectively; Fig. 7 and Table 7), and relatively small changes in proportions for wetter regions (by − 76 to − 6% and − 12 to + 19%). Irrigated late chickpea attained the greatest increase in profit and farm area (+ 301% and + 77%, Fig. 7), whereas irrigated early maize had the highest reduction (− 63% and − 51%), both at the drier regions. The substantial gain in returns (+ 301%) for irrigated late chickpea at the drier regions was driven by the high allocation of farm area (+ 77%; Fig. 7 and Table 7) to the most profitable farm system under future climates. Smallest changes in crop profit and farm area at the wetter regions were achieved by rainfed early chickpea (− 19% and − 5% respectively; Fig. 7 and Table 7). Minor declines (changes) in crop profits (− 19%; Fig. 7 and Table 7) at wetter regions was underpinned by slight reductions in area sown (− 5%) to economically viable crops under future climates relative to historical period. Rainfed early chickpea was only profitable at the wetter region of Hagley under both historical and future climates (Fig. 7).

Overall, irrigated early maize had the highest decrease in profit and cropping area (− 34% and − 29% respectively; Table 7) across regions under future climates, suggesting maize will be economically viable but most vulnerable to the detrimental impacts of climate change. Changes in cropping areas were an output of *WaterCan Profit*. Chickpeas attained the largest increase in average profit and cropping area (+ 65% and + 25%) across regions, projecting that chickpea will be the most climate-resilient profitable crops across the regions. Durum wheat was the least profitable crop (0.01 M, Fig. 7 and Table 7 averaged across regions) which showed no change in profit and area sown under both historical and future climates, indicating durum wheat will be unprofitable and less affected by the profound impacts of climate change.

At the whole farm scale, cool temperate wetter regions (e.g., Hagley) attained the lowest decline in average profits (by − 11%, Fig. 8) attributed to the high number of profitable crops (5 crops) whereas, the highest profit reduction (− 23%,) was at the moderate rainfall (wet) region (e.g., Frances) due to few profitable farm systems (1 system). Drier regions at Kerang and Griffith had moderate profit reductions (− 12% and − 19%), each with two profitable crops. Across all regions, crop profits decreased (− 8%, Table 7) while area planted marginally increased (+ 2%). Profit reductions were greater for dryland environments (− 29%) partly due to decrease in area sown (− 8%), whereas for irrigated scenarios profits reductions were modest (− 5%). In general, profits and farm area under irrigation increased (+ 24% and + 14%) at the drier regions and decreased (− 28% and − 5%) at the wetter regions. Area sown to different crop types across regions was estimated in *WaterCan Profit* based on all of the factors influencing profitability. Higher percentages of farm area under future climates were allocated to the most economically feasible crops (mostly irrigated enterprises); smaller portions of farm area were assigned to less profitable crops (mainly rainfed crops); these percentages were calculated by *WaterCan Profit* accounting for crop yields, water use, variable costs, water cost and grain price.

## Discussion

The aim of this study was to illustrate how the decision-support tool *WaterCan Profit* (WCP) can be used to determine how profit and whole farm crop combinations, including how profitability and whole farm systems will be impacted by and change under future climates. Even though Australian farms are exposed to the greatest climate volatility in the world, most previous studies have primarily examined climate change assuming no change in extreme weather events (e.g. Phelan et al.^43^). In the present study, we demonstrated how *WaterCan Profit* can be used to examine the impact of climate change on productivity and profitability, as well as how the distribution of most profitable crops changes over the whole farm as global warming intensifies. We demonstrate this framework using the five most important crops in Australia (barley, maize, wheat, chickpea, canola) in all of the major irrigation zones in Australia and thus we have confidence that our work is relevant to the majority of the irrigated sector in Australia. As well, and perhaps more importantly, the concepts and framework we demonstrate could be applied to any number of crops or genotypes, for any location in the world and under any climate horizon. The concepts shown here are thus generic and universally scalable. Future studies using WaterCan Profit could examine isolated aspects of agronomy, such as how irrigation management impacts on crop yields translate to economic outcomes at the whole farm scale. Such studies could also consider changes in future water supply under climate change; *WaterCan Profit* is ideally designed to examine how changes annual farm water allocation quantities may impact on productivity and profitability.

To advance the scientific endeavour and reliability under which yields are impacted by future climates, we build on pioneering methods developed by Harrison et al.^39^, to account for extreme events under future climates on top of background changes in temperature and rainfall. We found that shifts in OFPs caused by future climates were relatively higher under dryland conditions (23% and 39 days) than under irrigation (16% and 16-days respectively, see Figs. 4, S1, S2, S3 and S4), suggesting that irrigation partially mitigates the impact of climate change and more frequent extreme events. Our results show that irrigation indirectly mitigates the impacts of climate change on productivity by preventing the warming-induced decline in crop lifecycle that occurs for dryland crops. Thus, irrigation mitigates the extent to which development is increased as the climate warms and consequently the longer crop growing cycle allows greater biomass production and seed set. This result is analogous to findings of Schauberger et al.^76^, and Muleke et al.^11^. Our study also found that yield reductions were higher in the low rainfall (‘dry and hot’) regions of Griffith (41%) and Kerang (39%, Figs. 4, S1, S2, S3 and S4), partly because these regions experienced the highest temperature rise in future climates on top of already low prevailing rainfall (Fig. 2), which truncated further crop lifecycles. These findings are consistent with other climate change yield projections^54,77^ which have suggested that yield loss may be greater in regions that are already dry (compared with those with higher rainfall, such as coastal regions in Australia). The yield reductions induced by climate change in our present study substantially impacted crop GMs and profitability for future climates in most of the regions.

Our results also reveal that future climates reduced GMs by -21% and the number of profitable farm systems by 3–5 (Figs. 5, 6, Tables 6, and S5). The average GM reduction was greater for dryland scenarios (− 11 to 24%) compared with irrigated conditions (− 4 to 14%) under future climates, implying that irrigation partially mitigates the detrimental impacts of climate change on crop GMs, and represents a potential adaptation for enhancing the resilience of the GMs to climate change. Our results agree with findings of Ghahramani et al.^78^, and Ghahramani et al.^79^, who reported GM declines of − 12% when using existing technology and management practices in 2030 climate. Collectively these findings suggest that farmers must innovate beyond current incremental adaptations (e.g. sowing time, N fertilizer, heat tolerant genetics and irrigation; Langworthy et al.^80^) to negate detrimental impacts of 2080 climate on GMs in Australia. One way this could be done might be greater adjustment in management between years based on predicted seasonal climate forecasts (viz.^81^ wherein farmers could substantially reduce inputs and cropped areas under forecasts of very dry seasons, and significantly intensify crop production under forecasts of high rainfall. This approach would be expected to yield greater benefits in high rainfall years (e.g., La Nina) and mitigate detrimental impacts in poor years, such as drought or extreme heat during flowering. This approach would dovetail with expected increased in farm profitability under future climates observed by others (− 27% to + 30% observed by Hughes and Gooday^82^). It is however worth noting that at the time of writing, the majority of grain producers in Australia perceive seasonal climate forecasts as too unreliable to use^83^, suggesting a need for improvement in short-term climatic forecasts in Australia.

We found that higher crop prices within the decision-support tool WCP generated the most profitable outcomes across regions and climates (Table 7), suggesting higher crop prices in-part cushion the impacts of climate change on farm profits. Although for grain consumers including the intensive livestock sector, such higher prices add additional costs, for grain farmers unique periods of markedly high prices often provide windows of opportunity to optimise farm profits which buffer climate and price risk exposure during subsequent leaner years. These observations agree with results of Hughes and Gooday^82^ and Hughes et al.^84^, who projected increments in grain prices by + 1% to + 29% under 2050 climates. A plausible option to minimise the negative effects of high prices for consumers would be the expansion in grain storage capacity (or grain import supply chains) to limit grain shortages during drought years^84^.

We also showed that climate change reduced the number of economically viable farm systems (by 1–3) across regions (Fig. 7, and Table 7). The reduction was higher at the drier regions (− 3 crops) compared to the wetter regions (− 1 crops). The greater decline in profitable farm systems at drier regions was partly attributed to climate-related yield losses by majority of the crops. This result suggests that climate change will greatly limit profitable crop enterprises at the drier regions, reflecting a potential for farmers to shift away from crop enterprises (to either livestock^78^ and mixed farming enterprises^84^ or increase crop enterprises at the wetter regions^85^).

Our results show that whole-farm profit losses due to climate change were highest at the moderate rainfall (wet) regions (ca. − 23%) and lower at the wetter regions (ca. − 11%, Fig. 8 and Table 7). We found severe to moderate profit reductions at the drier regions (− 12% to − 19%). The substantial financial losses at the wet regions were implicated to the additional irrigation water costs and the low diversity of profitable crop portfolios (since only irrigated chickpeas was economically viable at the wet regions). These results agree with findings reported by Connor et al.^86^, who showed that increased water cost in addition to climate-induced yield reductions contributed to a precipitous decline in profits at moderate rainfall regions of South Australia (by − 22%) compared with drier regions of Victoria (by − 9%) under mild climate change scenarios in 2030. Analogous to our findings on impact of diversification of cropping options, Viguier et al.^87^, showed that whole-farm economic performance for non-diversified (mono-cropping) systems decreased considerably (− 59 ± 26%) in comparison with diversified (multi-cropping) systems (− 35 ± 8%) across five arable production regions of France. Together, these results demonstrate that sustainable gains in farm profit under future climates will not depend entirely on rainfall gradient to offset climate-related economic losses but rather, gains are a function of a complex interplay between crop price, yield, variable costs, water costs and innovative adaptations (see Figs. 7, 8 and 9).

The minimal climate-induced economic losses at the wetter regions (e.g., Hagley) shown here can be attributed to increased diversity of profitable crops (+ 5 crops, Fig. 8 and Table 7) in addition to relatively low climatic yield penalty (Figs. 4, S1, S2, S3 and S4), which aligns well with the observation that diversification of crop incomes provides a viable pathway for enhancing the resilience of whole-farm profits by spreading the economic risks posed by climate change^87–93^.

Our projections show that climate change may cause induce major shifts in crop profits and area able to be sown in drier regions (by − 63 to + 301% and − 51 to + 77% respectively; Fig. 7 and Table 7) but cause smaller changes in wetter regions (− 76 to − 6% and − 12 to + 19%, respectively). The largest shifts under future climates were away from crops with higher water use and lower grain price (e.g. maize) and towards crops with lower yields, higher price per tonne and lower water use (chickpeas). These results are broadly consistent with the recent literature. For example, Ghahramani et al.^78^, projected greater changes in profits at the drier regions (− 74% to + 16%) compared to wetter regions (− 15% to − 10%) across Western Australia, South Australia and New South Wales under severe climate change scenarios in 2030. Hughes et al.^84^, also simulated larger changes in profits in the drier regions of Western Australia (− 98% to − 9%) compared with more modest changes at wetter regions in Tasmania (− 13% to − 7%) under 2050 climates.

Our analyses show that enterprises with chickpea crops had the largest increase in average profit and cropping area under future climates (+ 65% and + 25% respectively; Fig. 7 and Table 7) in most of the regions, suggesting that chickpeas are a climate-resilient and profitable crop due to high commodity prices and relatively low water requirements, which together buffer the negative climate change impacts on yield. Irrigated early maize will be economically viable but characterised by sharp declines in profit and area planted (− 34% and − 29% respectively; Table 7), implying maize will be profitable but more vulnerable to climate change as lower maize prices and higher water requirements predispose profitability to climate-induced penalties (Fig. 9 and Table 7). Despite high potential yields, durum wheat will be most unprofitable and unresponsive to climate change in most of the regions, mainly due to lower grain prices. More broadly, these results highlight the potential for farmers to shift towards climate-resilient profitable grain-legumes (e.g., chickpeas) and away from less economically viable dominant cereals (e.g., durum wheat and barley) under future climates. These findings are congruent with previous work^9^ which suggest that legumes such as mungbeans and chickpeas would be most profitable options when income uncertainties are taken into account. Potential future surge in production and supply of grain-legumes at the expense of cereals would exert significant pressure on demand and supply dynamics at global and domestic levels^94,95^, resulting in decreased legume prices^96^ and spikes in prices for cereals^97,98^ and cereal end-products (e.g., durum-based pasta, Gal^95^, Freebairn^99^).

Our results reveal that average crop profits decreased (− 8%, Table 7) under future climates. Profit losses were greater in dryland environments (− 29%) attributed to decline in area sown (− 8%) and more severe yield losses (− 23% Tables 5, S1, S2, S3 and S4). Irrigated scenarios experienced modest reductions in profit (− 5%) partly due to increased area sown on farm (+ 4%) and less severe yield reductions (− 16%). We found that profitable rainfed crops constituted 20% of farm area at the wetter regions, thus contributing to the decline in profits and farm area under irrigation (− 28% and − 5%, Table 7). In contrast, profitable crops in drier zones were primarily irrigated, resulting in greater incremental increases in profits and cropping area under irrigation (+ 24% and + 14%). Our results also suggest irrigating greater areas of the farm in drier regions partially compensates for detrimental climate change impacts on farm profits. These results are consistent with findings of Elliott et al.^100^. However, a key challenge for future irrigated crops will be the reduction in freshwater availability^100^.

As for any study, this work had some limitations. Our simulations of crop yield considered only the impacts of frost, heat and water stress, we did not consider impacts of other projected changes in abiotic (e.g., waterlogging) or biotic (e.g., weeds, pests and diseases) stresses. Our study also uses climate projections defined by the current state of the art of GCMs. While such projections may change or improve in future, we can only use the available forecasts that we have at the time of writing. Another deliberate assumption of the present study was that full irrigation was applied, because we did not wish to confound changes in water stress due to suboptimal management with other changes caused by global warming, e.g. enhanced rate of crop development. Future studies may wish to take into account regional factors influencing farm-scale irrigation supply and impose these changes on the future farming system.

## Conclusions

We assessed the collective and interacting impact of meteorological, biophysical and economic factors on whole-farm profit and crop options under climate change using decision-support tool *WaterCan Profit.* We revealed that farmers with less diverse crop types at their disposal and higher irrigation variable costs will likely suffer the greatest climate-related financial losses (ca. − 23%). Nevertheless, use of irrigation *per* se was shown to increase profits and cropping area (+ 24% and + 14% respectively) in the drier regions, suggesting that irrigation can be a viable adaptation to compensate detrimental climate change impacts on farm profits. We showed that effects of climate change on whole farm profit were not related to prevailing climate type of the region, with future climates depressing profit by 11–23% relative to historical climates. The climate-induced whole farm economic losses were closely linked to decline in area sown (− 8%) and more severe yield penalties (− 23%). Impacts of future climates were more closely related to crop type and maturity duration; indeed, many crop types that were traditionally profitable under historical climates were no longer profitable in future. We suggest that future work on drought adaptation use genotypic selection criteria more diverse than yield alone. We conclude that crop types with (1) higher value per unit weight, (2) lower water requirements per land area and (3) higher water-use efficiency are more likely to ensure the sustainability and prosperity of irrigated grain production systems.

## Supplementary Information

Supplementary Information.

## Data Availability

The data that support the findings of this study are available from the corresponding author, [Matthew Tom Harrison], upon reasonable request.
