# Supplementary material for: Whole farm planning raises profit despite burgeoning climate crisis
Source: Sci Rep. 2022 Oct 13;12:17188. doi: 10.1038/s41598-022-20896-z (PMC9562302; doi:10.1038/s41598-022-20896-z)
Supplement: Supplementary file 1 — Supplementary Information. [file 41598_2022_20896_MOESM1_ESM.docx]

**Supplementary Information**


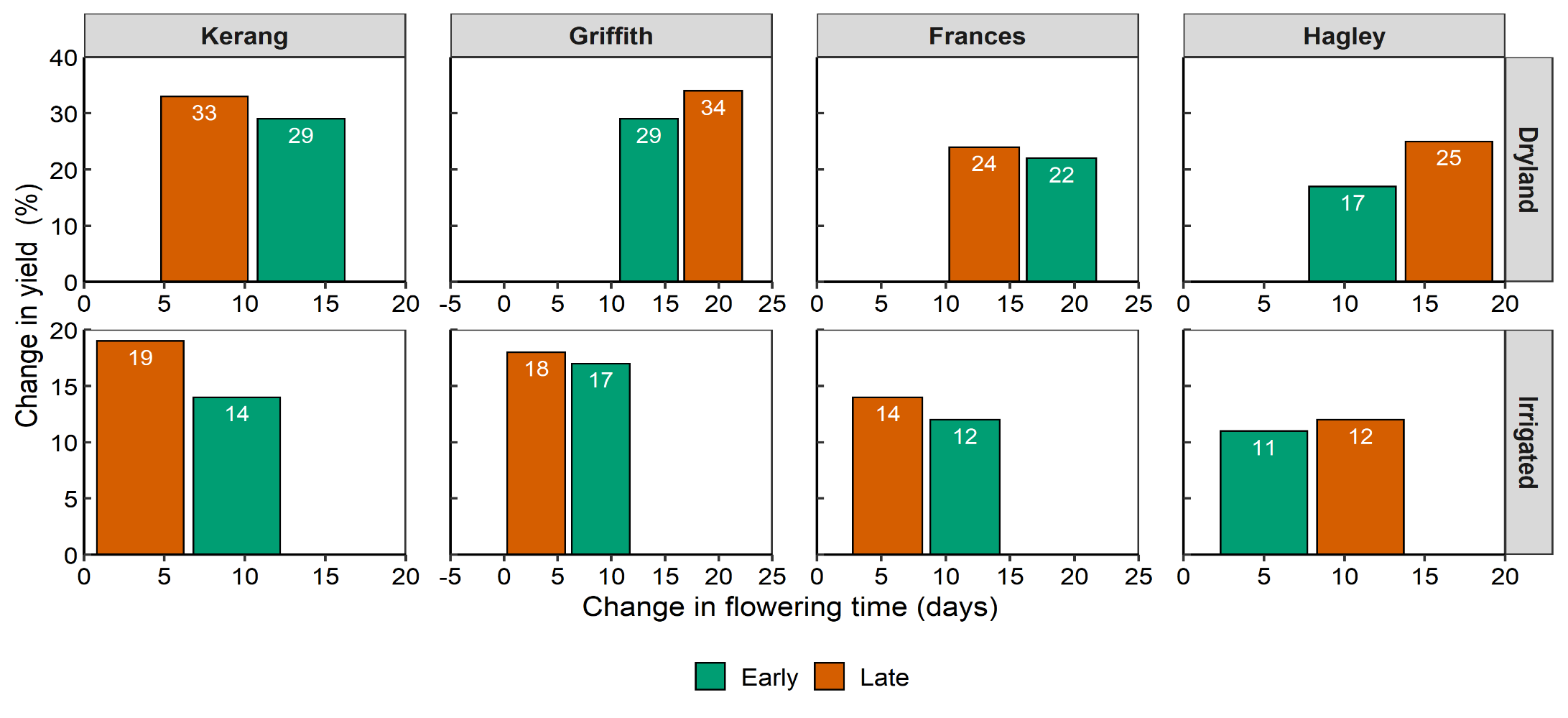


**Fig S1 Impacts of future climates on optimal flowering times** **and yield.** The columns show relationships between shifts in optimal flowering duration (in days) and percentage reduction in peak yield for early (green columns) and late (brown columns) genotypes of spring barley in dryland (top row) and irrigated (bottom row) conditions across a range of representative environments in Australian irrigated cropping regions. Future (2070-2089) climates truncated crop lifecycles, shifting forward flowering times relative to historical (1985-2004) climates. Irrigation partially off-set the forward shifts in flowering by lengthening lifecycles for irrigated crops resulting in later flowering periods in some regions (indicated by negative values). Regions are depicted along a rainfall gradient, from the lowest average annual rainfall (Kerang, 387 mm) to the highest (Hagley, 680 mm).

**
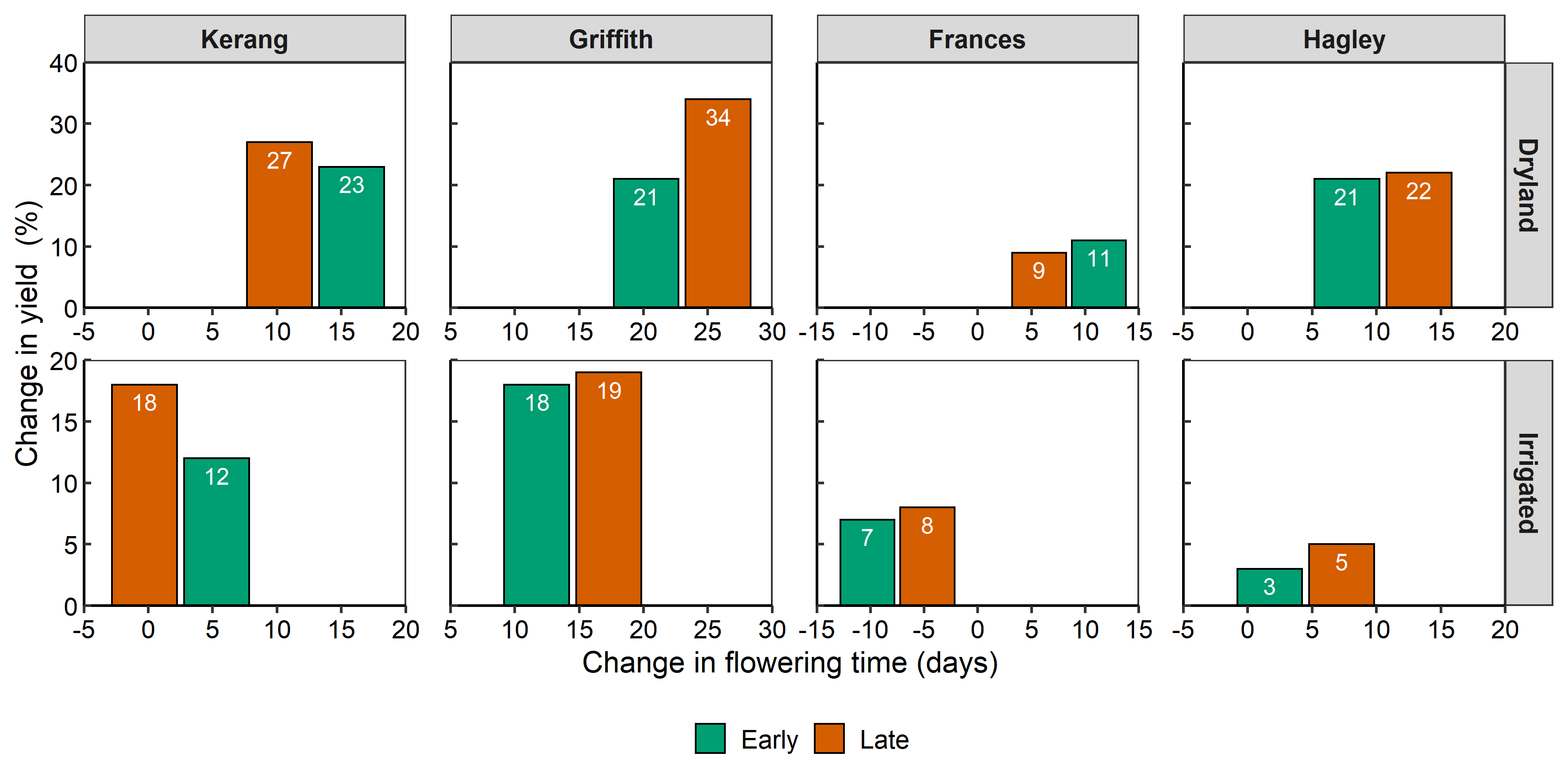
**

**Fig S2** **Impacts of future climates on optimal flowering times** **and yield.** The columns show relationships between shifts in optimal flowering duration (in days) and percentage reduction in peak yield for early (green columns) and late (brown columns) genotypes of canola in dryland (top row) and irrigated (bottom row) conditions across a range of representative environments in Australian irrigated cropping regions. Future (2070-2089) climates truncated crop lifecycles, shifting forward flowering times relative to historical (1985-2004) climates. Irrigation partially off-set the forward shifts in flowering by lengthening lifecycles for irrigated crops resulting in later flowering periods in some regions (indicated by negative values). Regions are depicted along a rainfall gradient, from the lowest average annual rainfall (Kerang, 387 mm) to the highest (Hagley, 680 mm).


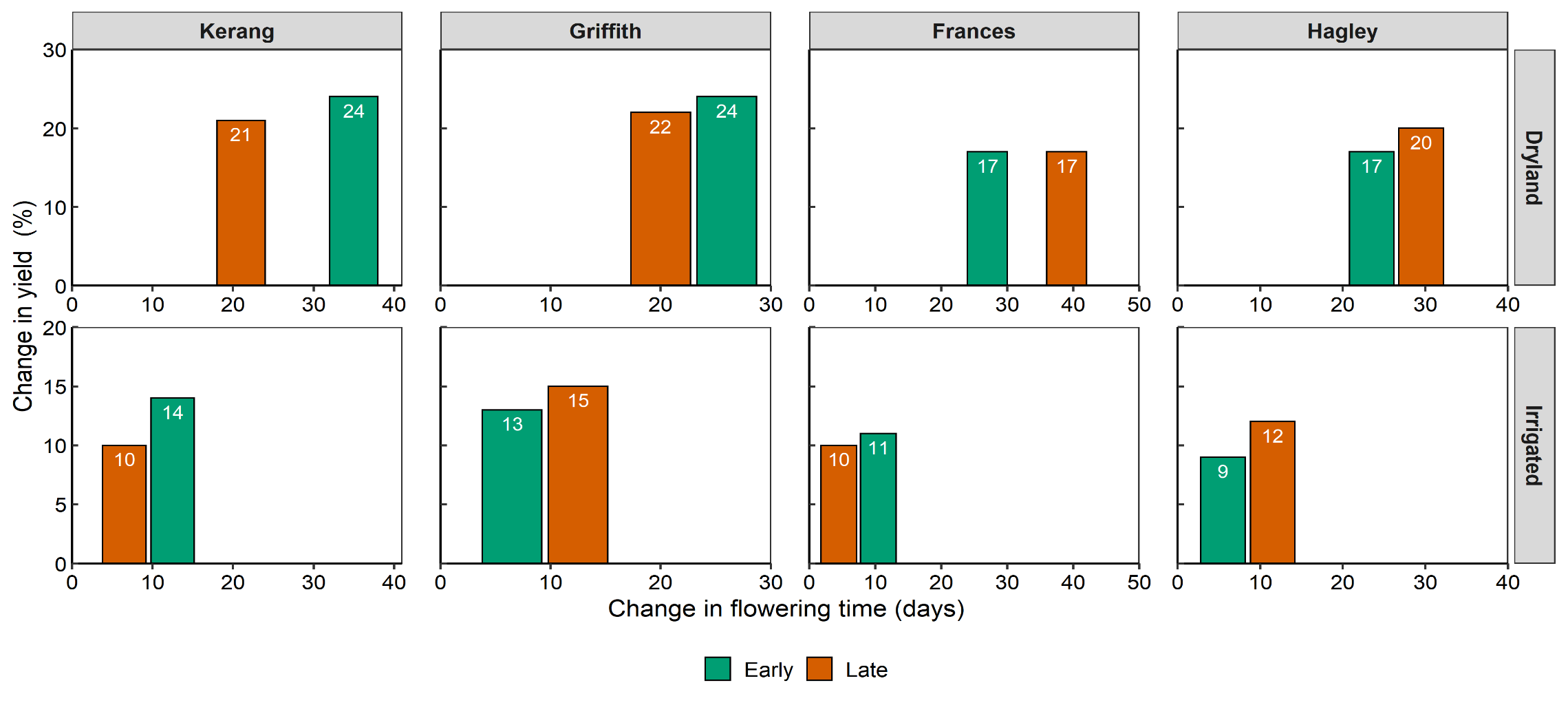


**Fig S3 Impacts of future climates on optimal flowering times** **and yield.** The columns show relationships between shifts in optimal flowering duration (in days) and percentage reduction in peak yield for early (green columns) and late (brown columns) genotypes of chickpeas in dryland (top row) and irrigated (bottom row) conditions across a range of representative environments in Australian irrigated cropping regions. Future (2070-2089) climates truncated crop lifecycles, shifting forward flowering times relative to historical (1985-2004) climates. Irrigation partially off-set the forward shifts in flowering by lengthening lifecycles for irrigated crops (shown in the bottom row). Regions are depicted along a rainfall gradient, from the lowest average annual rainfall (Kerang, 387 mm) to the highest (Hagley, 680 mm).


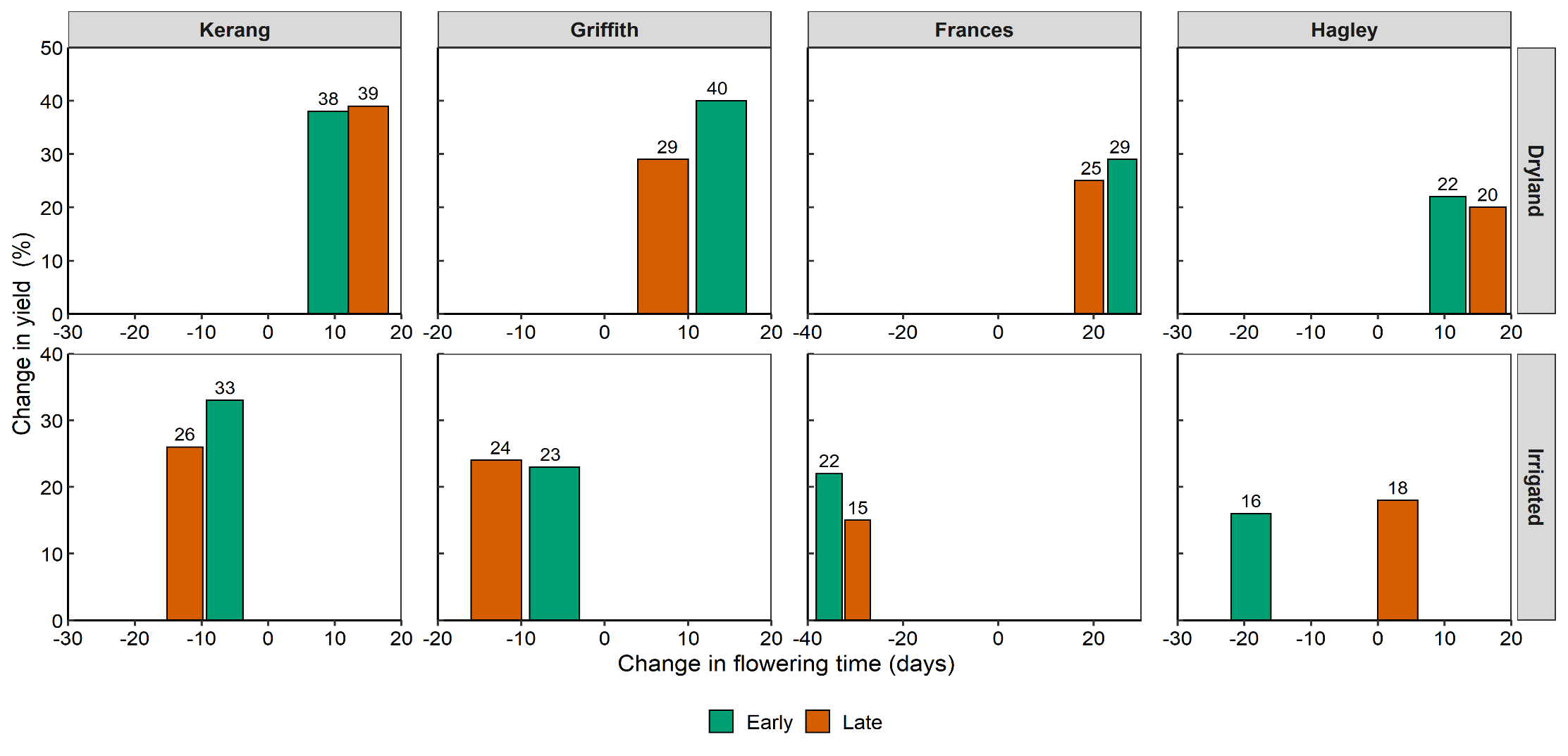


**Fig S4 Impacts of future climates on optimal flowering times** **and yield.** The columns show relationships between shifts in optimal flowering duration (in days) and percentage reduction in peak yield for early (green columns) and late (brown columns) genotypes of maize in dryland (top row) and irrigated (bottom row) conditions across a range of representative environments in Australian irrigated cropping regions. Future (2070-2089) climates truncated crop lifecycles, shifting forward flowering times relative to historical (1985-2004) climates. Irrigation partially off-set the forward shifts in flowering by lengthening lifecycles for irrigated crops resulting in later flowering periods in most of the regions (indicated by negative values). Regions are depicted along a rainfall gradient, from the lowest average annual rainfall (Kerang, 387 mm) to the highest (Hagley, 680 mm).


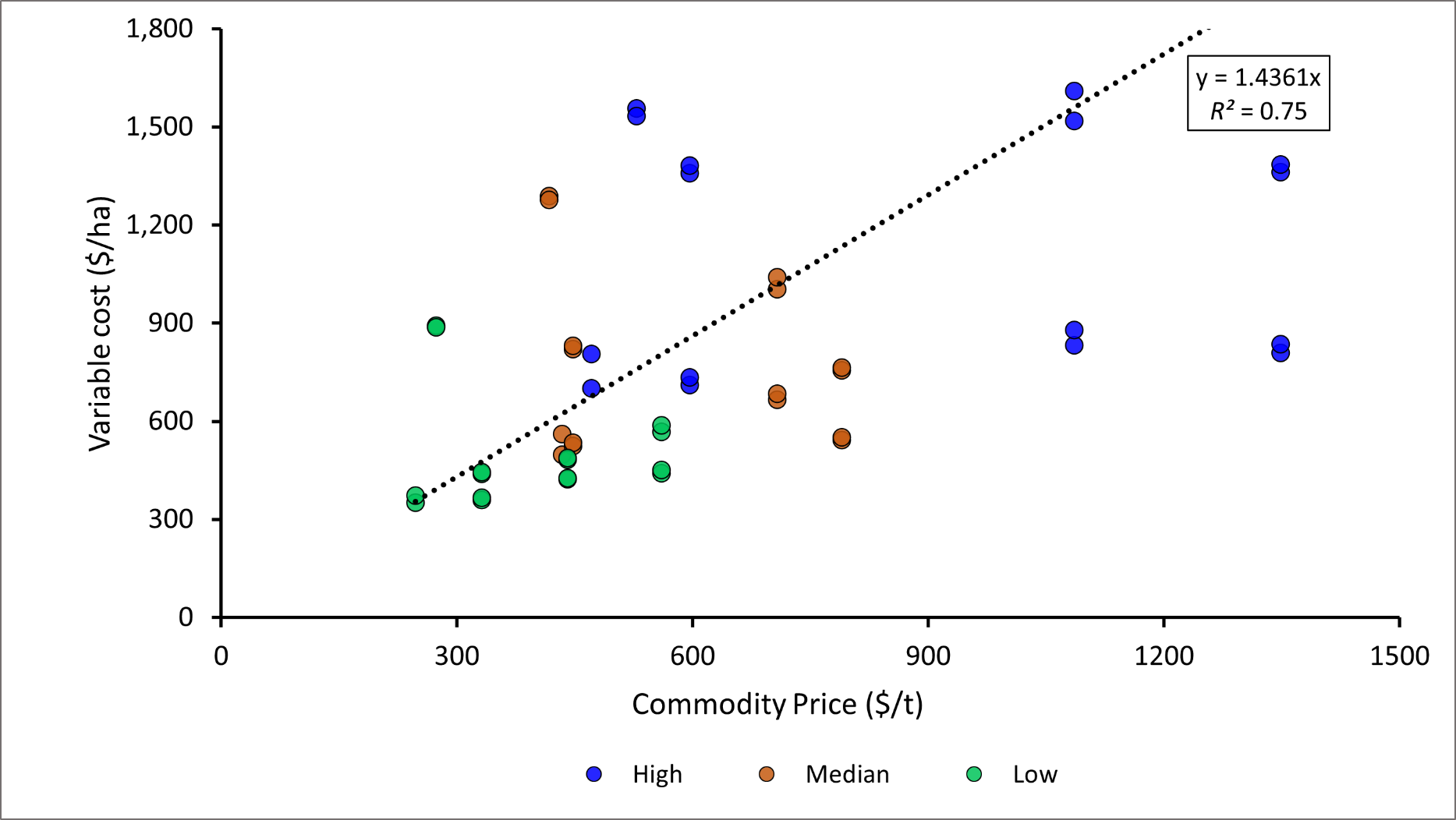


**Fig S5** Correlation between the low (green), median (orange), and high (blue) variable costs per hectare ($/ha) and commodity prices per tonne ($/t) for early and genotypes of chickpeas, canola, maize barley and wheat in dryland and irrigated conditions across a range of representative environments in Australian irrigated cropping regions under historical (1985-2004) and future (2070-2089) climates.

**Table S1** **Yield, optimal flowering periods and average water applied per annum** of irrigated and dryland spring barley across a range of representative environments in Australian irrigated cropping regions under historical (H = 1985-2004) and future (F = 2070-2089) climates. Crop duration denotes earliest sowing to start of flowering.

| **Region** | **Genotype** | **Regime** | **Period** | **Optimal range of sowing dates** | | **Maximum Yield** | **Optimal range of flowering period** | | **Crop Duration** | **Average irrigation per year** |
| --- | --- | --- | --- | --- | --- | --- | --- | --- | --- | --- |
|  |  |  |  | **Earliest** | **Latest** | **(kg/ha)** | **Start** | **Close** | **(Days)** | **(ML)** |
| Kerang | Early | Dryland | H | 24-May | 7-Jun | 3,680 | 31-Aug | 17-Sep | 99 | - |
|  |  |  | F | 31-May | 7-Jun | 2,637 | 20-Aug | 6-Sep | 81 | - |
|  |  | Irrigated | H | 28-Jun | 5-Jul | 5,624 | 1-Oct | 7-Oct | 95 | 655 |
|  |  |  | F | 14-Jun | 5-Jul | 4,871 | 24-Sep | 28-Sep | 102 | 642 |
|  | Late | Dryland | H | 26-Apr | 10-May | 4,537 | 29-Aug | 14-Sep | 125 | - |
|  |  |  | F | 3-May | 10-May | 3,047 | 19-Aug | 4-Sep | 108 | - |
|  |  | Irrigated | H | 24-May | 5-Jul | 8,466 | 18-Oct | 28-Oct | 147 | 866 |
|  |  |  | F | 7-Jun | 28-Jun | 6,902 | 12-Oct | 18-Oct | 127 | 839 |
| Griffith | Early | Dryland | H | 31-May | 28-Jun | 3,478 | 2-Sep | 21-Sep | 94 | - |
|  |  |  | F | 31-May | 7-Jun | 2,496 | 17-Aug | 31-Aug | 78 | - |
|  |  | Irrigated | H | 21-Jun | 5-Jul | 5,902 | 25-Sep | 5-Oct | 96 | 946 |
|  |  |  | F | 7-Jun | 5-Jul | 4,936 | 18-Sep | 28-Sep | 103 | 921 |
|  | Late | Dryland | H | 10-May | 24-May | 3,904 | 4-Sep | 27-Sep | 117 | - |
|  |  |  | F | 3-May | 10-May | 2,596 | 18-Aug | 2-Sep | 107 | - |
|  |  | Irrigated | H | 31-May | 21-Jun | 8,460 | 26-Sep | 22-Oct | 118 | 1,184 |
|  |  |  | F | 7-Jun | 28-Jun | 6,967 | 21-Sep | 15-Oct | 106 | 1,154 |
| Frances | Early | Dryland | H | 21-Jun | 5-Jul | 3,857 | 4-Oct | 12-Oct | 105 | - |
|  |  |  | F | 5-Jul | 5-Jul | 3,045 | 16-Sep | 1-Oct | 73 | - |
|  |  | Irrigated | H | 28-Jun | 5-Jul | 4,097 | 7-Oct | 22-Oct | 101 | 374 |
|  |  |  | F | 5-Jul | 5-Jul | 3,638 | 28-Sep | 11-Oct | 85 | 367 |
|  | Late | Dryland | H | 24-May | 21-Jun | 5,351 | 28-Sep | 29-Oct | 127 | - |
|  |  |  | F | 7-Jun | 7-Jun | 4,109 | 14-Sep | 20-Oct | 99 | - |
|  |  | Irrigated | H | 14-Jun | 5-Jul | 7,492 | 27-Oct | 4-Nov | 135 | 517 |
|  |  |  | F | 28-Jun | 5-Jul | 6,491 | 19-Oct | 23-Oct | 113 | 478 |
| Hagley | Early | Dryland | H | 28-Jun | 5-Jul | 4,489 | 15-Oct | 21-Oct | 109 | - |
|  |  |  | F | 5-Jul | 5-Jul | 3,739 | 2-Oct | 13-Oct | 89 | - |
|  |  | Irrigated | H | 5-Jul | 5-Jul | 4,945 | 18-Oct | 27-Oct | 105 | 659 |
|  |  |  | F | 5-Jul | 5-Jul | 4,419 | 11-Oct | 17-Oct | 98 | 654 |
|  | Late | Dryland | H | 24-May | 21-Jun | 6,795 | 22-Oct | 28-Oct | 151 | - |
|  |  |  | F | 24-May | 7-Jun | 5,163 | 8-Oct | 22-Oct | 137 | - |
|  |  | Irrigated | H | 21-Jun | 5-Jul | 9,804 | 5-Nov | 14-Nov | 137 | 846 |
|  |  |  | F | 5-Jul | 5-Jul | 8,669 | 27-Oct | 5-Nov | 114 | 820 |

**Table S2: Yield, optimal flowering periods and average water applied per annum** of irrigated and dryland canola across a range of representative environments in Australian irrigated cropping regions under historical (H = 1985-2004) and future (F = 2070-2089) climates. Crop duration denotes earliest sowing to start of flowering.

| **Region** | **Genotype** | **Regime** | **Period** | **Optimal range of sowing dates** | | **Maximum Yield** | **Optimal range of flowering period** | | **Crop Duration** | **Average irrigation per year** |
| --- | --- | --- | --- | --- | --- | --- | --- | --- | --- | --- |
|  |  |  |  | **Earliest** | **Latest** | **(kg/ha)** | **Start** | **Close** | **(Days)** | **(ML)** |
| Kerang | Early | Dryland | H | 26-Apr | 10-May | 2,207 | 22-Jul | 9-Aug | 87 | - |
|  |  |  | F | 19-Apr | 24-May | 1,717 | 8-Jul | 1-Aug | 80 | - |
|  |  | Irrigated | H | 10-May | 28-Jun | 3,480 | 4-Aug | 30-Aug | 86 | 996 |
|  |  |  | F | 17-May | 31-May | 3,063 | 1-Aug | 20-Aug | 76 | 1,128 |
|  | Late | Dryland | H | 5-Apr | 19-Apr | 2,954 | 24-Jul | 12-Aug | 110 | - |
|  |  |  | F | 5-Apr | 26-Apr | 2,184 | 12-Jul | 23-Jul | 98 | - |
|  |  | Irrigated | H | 12-Apr | 17-May | 4,871 | 30-Jul | 5-Sep | 109 | 1,422 |
|  |  |  | F | 19-Apr | 26-Apr | 4,002 | 28-Jul | 18-Aug | 100 | 1,625 |
| Griffith | Early | Dryland | H | 10-May | 7-Jun | 1,931 | 2-Aug | 25-Aug | 84 | - |
|  |  |  | F | 26-Apr | 17-May | 1,532 | 11-Jul | 22-Jul | 76 | - |
|  |  | Irrigated | H | 24-May | 21-Jun | 3,662 | 9-Aug | 1-Sep | 77 | 1,405 |
|  |  |  | F | 10-May | 24-May | 3,029 | 27-Jul | 3-Aug | 78 | 1,633 |
|  | Late | Dryland | H | 19-Apr | 3-May | 2,632 | 9-Aug | 20-Aug | 112 | - |
|  |  |  | F | 5-Apr | 19-Apr | 1,757 | 16-Jul | 30-Jul | 102 | - |
|  |  | Irrigated | H | 19-Apr | 31-May | 5,003 | 13-Aug | 6-Sep | 116 | 1,971 |
|  |  |  | F | 12-Apr | 26-Apr | 4,074 | 28-Jul | 6-Aug | 107 | 2,289 |
| Frances | Early | Dryland | H | 3-May | 31-May | 2,424 | 5-Aug | 16-Aug | 94 | - |
|  |  |  | F | 17-May | 7-Jun | 2,168 | 25-Jul | 7-Aug | 69 | - |
|  |  | Irrigated | H | 17-May | 5-Jul | 3,066 | 17-Aug | 24-Aug | 92 | 529 |
|  |  |  | F | 24-May | 28-Jun | 2,856 | 26-Aug | 2-Sep | 94 | 592 |
|  | Late | Dryland | H | 5-Apr | 3-May | 3,337 | 3-Aug | 18-Aug | 120 | - |
|  |  |  | F | 19-Apr | 26-Apr | 3,047 | 28-Jul | 12-Aug | 100 | - |
|  |  | Irrigated | H | 19-Apr | 5-Jul | 3,912 | 17-Aug | 8-Sep | 120 | 779 |
|  |  |  | F | 19-Apr | 17-May | 3,614 | 23-Aug | 26-Aug | 126 | 879 |
| Hagley | Early | Dryland | H | 10-May | 28-Jun | 2,712 | 26-Aug | 14-Sep | 108 | - |
|  |  |  | F | 24-May | 5-Jul | 2,162 | 17-Aug | 2-Sep | 85 | - |
|  |  | Irrigated | H | 14-Jun | 5-Jul | 3,701 | 14-Sep | 23-Sep | 92 | 920 |
|  |  |  | F | 28-Jun | 5-Jul | 3,593 | 10-Sep | 14-Sep | 74 | 1,032 |
|  | Late | Dryland | H | 19-Apr | 24-May | 3,598 | 28-Aug | 16-Sep | 131 | - |
|  |  |  | F | 26-Apr | 31-May | 2,821 | 16-Aug | 31-Aug | 112 | - |
|  |  | Irrigated | H | 10-May | 5-Jul | 5,488 | 25-Sep | 2-Oct | 138 | 1,328 |
|  |  |  | F | 21-Jun | 5-Jul | 5,220 | 20-Sep | 29-Sep | 91 | 1,596 |

**Table S3: Yield, optimal flowering periods and average water applied per annum** of irrigated and dryland chickpeas across a range of representative environments in Australian irrigated cropping regions under historical (H = 1985-2004) and future (F = 2070-2089) climates. Crop duration denotes earliest sowing to start of flowering.

| **Region** | **Genotype** | **Regime** | **Period** | **Optimal range of sowing dates** | | **Maximum Yield** | **Optimal range of flowering period** | | **Crop Duration** | **Average irrigation per year** |
| --- | --- | --- | --- | --- | --- | --- | --- | --- | --- | --- |
|  |  |  |  | **Earliest** | **Latest** | **(kg/ha)** | **Start** | **Close** | **(Days)** | **(ML)** |
| Kerang | Early | Dryland | H | 12-Apr | 3-May | 3,167 | 11-Jul | 12-Aug | 90 | - |
|  |  |  | F | 19-Apr | 26-Apr | 2,414 | 6-Jun | 25-Jul | 48 | - |
|  |  | Irrigated | H | 3-May | 7-Jun | 4,170 | 5-Aug | 16-Sep | 94 | 817 |
|  |  |  | F | 26-Apr | 17-May | 3,615 | 26-Jul | 14-Aug | 91 | 871 |
|  | Late | Dryland | H | 29-Mar | 19-Apr | 3,479 | 6-Jul | 12-Aug | 99 | - |
|  |  |  | F | 12-Apr | 19-Apr | 2,783 | 15-Jun | 24-Jul | 64 | - |
|  |  | Irrigated | H | 12-Apr | 7-Jun | 4,423 | 7-Aug | 18-Sep | 117 | 948 |
|  |  |  | F | 12-Apr | 19-Apr | 3,994 | 29-Jul | 4-Aug | 108 | 1,008 |
| Griffith | Early | Dryland | H | 19-Apr | 3-May | 2,986 | 15-Jul | 10-Aug | 87 | - |
|  |  |  | F | 12-Apr | 26-Apr | 2,298 | 21-Jun | 16-Jul | 70 | - |
|  |  | Irrigated | H | 3-May | 14-Jun | 3,993 | 27-Jul | 8-Sep | 85 | 1,084 |
|  |  |  | F | 26-Apr | 3-May | 3,490 | 18-Jul | 29-Jul | 83 | 1,216 |
|  | Late | Dryland | H | 12-Apr | 19-Apr | 3,231 | 15-Jul | 8-Aug | 94 | - |
|  |  |  | F | 12-Apr | 12-Apr | 2,528 | 23-Jun | 16-Jul | 72 | - |
|  |  | Irrigated | H | 19-Apr | 24-May | 4,334 | 22-Jul | 9-Sep | 94 | 1,239 |
|  |  |  | F | 12-Apr | 26-Apr | 3,727 | 12-Jul | 27-Jul | 91 | 1,388 |
| Frances | Early | Dryland | H | 17-May | 14-Jun | 3,471 | 21-Aug | 21-Sep | 96 | - |
|  |  |  | F | 3-May | 31-May | 2,915 | 25-Jul | 18-Aug | 83 | - |
|  |  | Irrigated | H | 31-May | 5-Jul | 4,044 | 9-Sep | 30-Sep | 101 | 483 |
|  |  |  | F | 17-May | 5-Jul | 3,604 | 30-Aug | 18-Sep | 105 | 500 |
|  | Late | Dryland | H | 3-May | 24-May | 3,710 | 27-Aug | 20-Sep | 116 | - |
|  |  |  | F | 19-Apr | 17-May | 3,096 | 19-Jul | 26-Aug | 91 | - |
|  |  | Irrigated | H | 10-May | 28-Jun | 4,311 | 5-Sep | 11-Oct | 118 | 568 |
|  |  |  | F | 10-May | 14-Jun | 3,884 | 31-Aug | 27-Sep | 113 | 582 |
| Hagley | Early | Dryland | H | 24-May | 5-Jul | 4,663 | 16-Sep | 4-Oct | 115 | - |
|  |  |  | F | 10-May | 14-Jun | 3,893 | 21-Aug | 21-Sep | 103 | - |
|  |  | Irrigated | H | 7-Jun | 5-Jul | 5,348 | 29-Sep | 14-Oct | 114 | 743 |
|  |  |  | F | 14-Jun | 5-Jul | 4,874 | 22-Sep | 6-Oct | 100 | 812 |
|  | Late | Dryland | H | 17-May | 14-Jun | 4,853 | 20-Sep | 13-Oct | 126 | - |
|  |  |  | F | 26-Apr | 24-May | 3,910 | 24-Aug | 24-Sep | 120 | - |
|  |  | Irrigated | H | 7-Jun | 5-Jul | 5,976 | 4-Oct | 29-Oct | 119 | 799 |
|  |  |  | F | 14-Jun | 5-Jul | 5,287 | 24-Sep | 19-Oct | 102 | 933 |

**Table S4: Yield, optimal flowering periods and average water applied per annum** of irrigated and dryland maize across a range of representative environments in Australian irrigated cropping regions under historical (H = 1985-2004) and future (F = 2070-2089) climates. Crop duration denotes earliest sowing to start of flowering.

| **Region** | **Genotype** | **Regime** | **Period** | **Optimal range of sowing dates** | | **Maximum Yield** | **Optimal range of flowering period** | | **Crop Duration** | **Average irrigation per year** |
| --- | --- | --- | --- | --- | --- | --- | --- | --- | --- | --- |
|  |  |  |  | **Earliest** | **Latest** | **(kg/ha)** | **Start** | **Close** | **(Days)** | **(ML)** |
| Kerang | Early | Dryland | H | 29-Dec | 29-Dec | 473 | 23-Feb | 3-Mar | 56 | - |
|  |  |  | F | 29-Dec | 12-Jan | 296 | 14-Feb | 1-Mar | 47 | - |
|  |  | Irrigated | H | 5-Jan | 5-Jan | 11,837 | 26-Feb | 16-Mar | 52 | 2,210 |
|  |  |  | F | 19-Jan | 19-Jan | 7,989 | 6-Mar | 15-Mar | 46 | 2,328 |
|  | Late | Dryland | H | 20-Oct | 20-Oct | 33 | 12-Jan | 13-Jan | 84 | - |
|  |  |  | F | 22-Sep | 22-Sep | 20 | 28-Dec | 4-Jan | 97 | - |
|  |  | Irrigated | H | 8-Dec | 8-Dec | 7,618 | 2-Mar | 27-Mar | 84 | 2,396 |
|  |  |  | F | 29-Dec | 29-Dec | 5,639 | 13-Mar | 26-Mar | 74 | 2,580 |
| Griffith | Early | Dryland | H | 12-Jan | 12-Jan | 982 | 27-Feb | 15-Mar | 46 | - |
|  |  |  | F | 19-Jan | 19-Jan | 596 | 13-Feb | 12-Mar | 25 | - |
|  |  | Irrigated | H | 12-Jan | 12-Jan | 11,263 | 29-Feb | 17-Mar | 48 | 2,830 |
|  |  |  | F | 19-Jan | 19-Jan | 8,755 | 6-Mar | 11-Mar | 47 | 3,057 |
|  | Late | Dryland | H | 22-Dec | 22-Dec | 127 | 8-Mar | 22-Mar | 76 | - |
|  |  |  | F | 29-Dec | 29-Dec | 91 | 1-Mar | 20-Mar | 62 | - |
|  |  | Irrigated | H | 22-Dec | 22-Dec | 7,464 | 5-Mar | 31-Mar | 73 | 3,134 |
|  |  |  | F | 5-Jan | 12-Jan | 5,685 | 18-Mar | 24-Mar | 72 | 3,410 |
| Frances | Early | Dryland | H | 15-Sep | 15-Sep | 217 | 27-Dec | 31-Dec | 103 | - |
|  |  |  | F | 22-Sep | 22-Sep | 156 | 1-Dec | 17-Dec | 70 | - |
|  |  | Irrigated | H | 17-Nov | 24-Nov | 5,884 | 25-Jan | 28-Feb | 69 | 1,756 |
|  |  |  | F | 12-Jan | 12-Jan | 4,631 | 1-Mar | 10-Mar | 48 | 1,887 |
|  | Late | Dryland | H | 15-Sep | 19-Jan | 159 | 24-Apr | 5-May | 221 | - |
|  |  |  | F | 15-Sep | 19-Jan | 119 | 5-Apr | 24-Apr | 202 | - |
|  |  | Irrigated | H | 22-Sep | 6-Oct | 3,483 | 28-Jan | 28-Feb | 128 | 1,577 |
|  |  |  | F | 8-Dec | 8-Dec | 2,988 | 27-Feb | 6-Mar | 81 | 1,914 |
| Hagley | Early | Dryland | H | 15-Sep | 15-Sep | 4,242 | 2-Jan | 5-Jan | 109 | - |
|  |  |  | F | 15-Sep | 22-Sep | 3,328 | 22-Dec | 30-Dec | 98 | - |
|  |  | Irrigated | H | 15-Sep | 6-Oct | 21,968 | 3-Jan | 25-Jan | 110 | 2,110 |
|  |  |  | F | 17-Nov | 24-Nov | 18,620 | 22-Jan | 2-Feb | 66 | 2,668 |
|  | Late | Dryland | H | 15-Sep | 15-Sep | 305 | 22-Feb | 22-Feb | 160 | - |
|  |  |  | F | 15-Sep | 6-Oct | 244 | 6-Feb | 17-Feb | 144 | - |
|  |  | Irrigated | H | 15-Sep | 15-Sep | 8,874 | 31-Jan | 6-Feb | 138 | 1,529 |
|  |  |  | F | 29-Sep | 13-Oct | 7,295 | 28-Jan | 2-Feb | 121 | 2,348 |

**Table S5:** The most profitable crops and the corresponding gross margins, yields, real prices and variable costs across a range of representative environments in Australian irrigated cropping regions under historical (H = 1985-2004) and future (F = 2070-2089) climates.

| **Region** | **Period** | **Rank** | **Crop** | **Genotype** | **Regime** | **Yield (t/ha)** | | | **Net sale price ($/t)** | | | **Total variable cost ($/ha)** | | | **Gross Margins ($/ha)** | | |
| --- | --- | --- | --- | --- | --- | --- | --- | --- | --- | --- | --- | --- | --- | --- | --- | --- | --- |
|  |  |  |  |  |  | **Low** | **Median** | **High** | **Low** | **Median** | **High** | **Low** | **Median** | **High** | **Low** | **Median** | **High** |
| Griffith | H | **1.** | Chickpea | Late | Irrigated | 1.3 | 2.2 | 4.3 | 441 | 790 | 1,348 | 520 | 813 | 1,384 | 54 | 899 | 4,460 |
|  |  | **2.** | Maize | Early | Irrigated | 3.4 | 5.6 | 11.3 | 273 | 418 | 528 | 930 | 1,311 | 1,567 | -7 | 1,040 | 4,385 |
|  |  | **3.** | Canola | Late | Irrigated | 1.7 | 2.7 | 5.2 | 560 | 708 | 1,086 | 616 | 1,149 | 1,648 | 354 | 785 | 4,033 |
|  |  | **4.** | Chickpea | Early | Irrigated | 1.2 | 2 | 4 | 441 | 790 | 1,348 | 515 | 805 | 1,356 | 13 | 772 | 4,028 |
|  |  | **5.** | Chickpea | Late | Rainfed | 1 | 1.6 | 3.2 | 441 | 790 | 1,348 | 423 | 544 | 815 | 4 | 732 | 3,541 |
|  |  | **6.** | Chickpea | Early | Rainfed | 0.9 | 1.5 | 3 | 441 | 790 | 1,348 | 420 | 538 | 799 | -25 | 642 | 3,228 |
|  |  | **7.** | Durum | Late | Irrigated | 2.3 | 3.6 | 7.1 | 332 | 448 | 596 | 480 | 877 | 1,408 | 267 | 750 | 2,818 |
|  |  | **8.** | Canola | Early | Irrigated | 1.3 | 2.1 | 3.9 | 560 | 708 | 1,086 | 596 | 1,084 | 1,560 | 149 | 375 | 2,665 |
|  |  | **9.** | Durum | Early | Irrigated | 2.1 | 3.3 | 6.5 | 332 | 448 | 596 | 473 | 865 | 1,360 | 214 | 629 | 2,509 |
|  |  | **10.** | Maize | Late | Irrigated | 2.2 | 3.7 | 7.5 | 273 | 418 | 528 | 925 | 1,298 | 1,536 | -313 | 260 | 2,408 |
|  | F | **1.** | Chickpea | Late | Irrigated | 1.1 | 1.9 | 3.7 | 441 | 790 | 1,348 | 520 | 813 | 1,384 | -27 | 659 | 3,641 |
|  |  | **2.** | Chickpea | Early | Irrigated | 1 | 1.7 | 3.5 | 441 | 790 | 1,348 | 515 | 805 | 1,356 | -54 | 574 | 3,350 |
|  |  | **3.** | Maize | Early | Irrigated | 2.6 | 4.4 | 8.8 | 273 | 418 | 528 | 930 | 1,311 | 1,567 | -212 | 516 | 3,059 |
|  |  | **4.** | Canola | Late | Irrigated | 1.2 | 2 | 4.1 | 560 | 708 | 1,086 | 616 | 1,149 | 1,648 | 69 | 293 | 2,775 |
|  |  | **5.** | Chickpea | Late | Rainfed | 0.8 | 1.3 | 2.5 | 441 | 790 | 1,348 | 423 | 544 | 815 | -89 | 454 | 2,593 |
|  |  | **6.** | Chickpea | Early | Rainfed | 0.7 | 1.1 | 2.3 | 441 | 790 | 1,348 | 420 | 538 | 799 | -116 | 370 | 2,300 |
| Frances | H | **1.** | Chickpea | Late | Irrigated | 1.3 | 2.2 | 4.3 | 441 | 790 | 1,348 | 494 | 737 | 1,231 | 76 | 965 | 4,581 |
|  |  | **2.** | Chickpea | Early | Irrigated | 1.2 | 2 | 4 | 441 | 790 | 1,348 | 491 | 731 | 1,210 | 44 | 866 | 4,242 |
|  |  | **3.** | Chickpea | Late | Rainfed | 1.1 | 1.9 | 3.7 | 441 | 790 | 1,348 | 431 | 557 | 853 | 60 | 909 | 4,149 |
|  |  | **4.** | Chickpea | Early | Rainfed | 1 | 1.7 | 3.5 | 441 | 790 | 1,348 | 428 | 551 | 835 | 31 | 820 | 3,845 |
|  |  | **5.** | Canola | Late | Irrigated | 1.4 | 2.2 | 4.1 | 560 | 708 | 1,086 | 535 | 895 | 1,362 | 252 | 653 | 3,135 |
|  |  | **6.** | Canola | Late | Rainfed | 1 | 1.7 | 3.3 | 560 | 708 | 1,086 | 457 | 696 | 898 | 104 | 485 | 2,724 |
|  |  | **7.** | Durum | Late | Irrigated | 1.9 | 3.1 | 6 | 332 | 448 | 596 | 443 | 738 | 1,201 | 193 | 641 | 2,364 |
|  |  | **8.** | Canola | Early | Irrigated | 1.1 | 1.8 | 3.3 | 560 | 708 | 1,086 | 522 | 872 | 1,306 | 122 | 376 | 2,272 |
|  |  | **9.** | Durum | Late | Rainfed | 1.6 | 2.6 | 4.9 | 332 | 448 | 596 | 375 | 550 | 767 | 156 | 593 | 2,171 |
|  |  | **10.** | Durum | Early | Irrigated | 1.8 | 2.8 | 5.4 | 332 | 448 | 596 | 435 | 727 | 1,175 | 147 | 530 | 2,064 |
|  | F | **1.** | Chickpea | Late | Irrigated | 1.2 | 1.9 | 3.9 | 441 | 790 | 1,348 | 494 | 737 | 1,231 | 19 | 797 | 4,005 |
|  |  | **2.** | Chickpea | Early | Irrigated | 1.1 | 1.8 | 3.6 | 441 | 790 | 1,348 | 491 | 731 | 1,210 | -14 | 693 | 3,649 |
|  |  | **3.** | Chickpea | Late | Rainfed | 0.9 | 1.5 | 3.1 | 441 | 790 | 1,348 | 431 | 557 | 853 | -21 | 666 | 3,320 |
|  |  | **4.** | Chickpea | Early | Rainfed | 0.9 | 1.5 | 2.9 | 441 | 790 | 1,348 | 428 | 551 | 835 | -43 | 600 | 3,095 |
|  |  | **5.** | Canola | Late | Irrigated | 1.1 | 1.8 | 3.6 | 560 | 708 | 1,086 | 535 | 895 | 1,362 | 73 | 385 | 2,562 |
|  |  | **6.** | Canola | Late | Rainfed | 0.9 | 1.5 | 3 | 560 | 708 | 1,086 | 457 | 696 | 898 | 55 | 383 | 2,410 |
| Hagley | H | **1.** | Maize | Early | Irrigated | 6.6 | 11 | 22 | 273 | 418 | 528 | 792 | 1,067 | 1,390 | 1,009 | 3,520 | 10,219 |
|  |  | **2.** | Chickpea | Late | Irrigated | 1.8 | 3 | 6 | 441 | 790 | 1,348 | 530 | 745 | 1,294 | 261 | 1,615 | 6,763 |
|  |  | **3.** | Chickpea | Early | Irrigated | 1.6 | 2.7 | 5.3 | 441 | 790 | 1,348 | 520 | 730 | 1,244 | 188 | 1,383 | 5,967 |
|  |  | **4.** | Chickpea | Late | Rainfed | 1.5 | 2.4 | 4.9 | 441 | 790 | 1,348 | 448 | 586 | 944 | 194 | 1,331 | 5,598 |
|  |  | **5.** | Chickpea | Early | Rainfed | 1.4 | 2.3 | 4.7 | 441 | 790 | 1,348 | 446 | 582 | 947 | 171 | 1,260 | 5,340 |
|  |  | **6.** | Canola | Late | Irrigated | 1.9 | 3 | 5.7 | 560 | 708 | 1,086 | 602 | 968 | 1,438 | 450 | 1,138 | 4,770 |
|  |  | **7.** | Durum | Late | Irrigated | 2.8 | 4.5 | 8.9 | 332 | 448 | 596 | 487 | 799 | 1,275 | 440 | 1,234 | 4,031 |
|  |  | **8.** | Durum | Early | Irrigated | 2.6 | 4.2 | 8.1 | 332 | 448 | 596 | 477 | 783 | 1,241 | 374 | 1,080 | 3,611 |
|  |  | **9.** | Maize | Late | Irrigated | 2.7 | 4.4 | 8.9 | 273 | 418 | 528 | 774 | 1,026 | 1,282 | -47 | 827 | 3,407 |
|  |  | **10.** | Canola | Late | Rainfed | 1.1 | 1.8 | 3.6 | 560 | 708 | 1,086 | 460 | 701 | 917 | 144 | 572 | 2,989 |
|  |  | **11.** | Canola | Early | Irrigated | 1.3 | 2.1 | 3.9 | 560 | 708 | 1,086 | 575 | 920 | 1,322 | 176 | 553 | 2,946 |
|  |  | **12.** | Durum | Late | Rainfed | 2 | 3.2 | 6.2 | 332 | 448 | 596 | 390 | 579 | 825 | 265 | 841 | 2,849 |
|  |  | **13.** | Durum | Early | Rainfed | 1.8 | 2.9 | 5.7 | 332 | 448 | 596 | 383 | 567 | 802 | 222 | 741 | 2,573 |
|  | F | **1.** | Maize | Early | Irrigated | 5.6 | 9.3 | 18.6 | 273 | 418 | 528 | 792 | 1,067 | 1,390 | 734 | 2,821 | 8,450 |
|  |  | **2.** | Chickpea | Late | Irrigated | 1.6 | 2.6 | 5.3 | 441 | 790 | 1,348 | 530 | 745 | 1,294 | 170 | 1,343 | 5,834 |
|  |  | **3.** | Chickpea | Early | Irrigated | 1.5 | 2.4 | 4.9 | 441 | 790 | 1,348 | 520 | 730 | 1,244 | 125 | 1,195 | 5,328 |
|  |  | **4.** | Chickpea | Late | Rainfed | 1.2 | 2 | 3.9 | 441 | 790 | 1,348 | 448 | 586 | 944 | 69 | 958 | 4,327 |
|  |  | **5.** | Chickpea | Early | Rainfed | 1.2 | 1.9 | 3.9 | 441 | 790 | 1,348 | 446 | 582 | 947 | 69 | 956 | 4,302 |
|  |  | **6.** | Canola | Late | Irrigated | 1.6 | 2.6 | 5.2 | 560 | 708 | 1,086 | 602 | 968 | 1,438 | 276 | 880 | 4,230 |
|  |  | **7.** | Durum | Late | Irrigated | 2.1 | 3.4 | 6.8 | 332 | 448 | 596 | 487 | 799 | 1,275 | 194 | 734 | 2,808 |
|  |  | **8.** | Durum | Early | Irrigated | 1.9 | 3.2 | 6.5 | 332 | 448 | 596 | 477 | 783 | 1,241 | 169 | 672 | 2,632 |
|  |  | **9.** | Canola | Early | Irrigated | 1.1 | 1.8 | 3.6 | 560 | 708 | 1,086 | 575 | 920 | 1,322 | 29 | 352 | 2,578 |
|  |  | **10.** | Maize | Late | Irrigated | 2.2 | 3.6 | 7.3 | 273 | 418 | 528 | 774 | 1,026 | 1,282 | -176 | 497 | 2,573 |

**Table S6:** Average annual allocation of irrigation water (ML) across a range of representative environments in Australian irrigated cropping regions under historical (1985-2004) and future (2070-2089) climates.

| Period | Region | Low | Median | High |
| --- | --- | --- | --- | --- |
| Historical | Griffith | 630 | 810 | 1100 |
|  | Kerang | 400 | 620 | 860 |
|  | Hagley | 380 | 530 | 720 |
|  | Frances | 150 | 420 | 500 |
| Future | Griffith | 670 | 870 | 1150 |
|  | Kerang | 450 | 650 | 880 |
|  | Hagley | 470 | 620 | 820 |
|  | Frances | 190 | 440 | 540 |

**Table S7:** Irrigation water application rates (ML/ha and mm) and prices ($/ML) for grain crops in the irrigation regions and zones across a range of representative environments in Australian irrigated cropping regions. Water prices were sourced from BoM (2021), ABARES (2021b) and Water Exchange (sourced from: <https://www.waterexchange.com.au/>).

| Region | Crop | Low | | Median | | High | | Price | Zone |
| --- | --- | --- | --- | --- | --- | --- | --- | --- | --- |
|  |  | **(ML/ha)** | **(mm)** | **(ML/ha)** | **(mm)** | **(ML/ha)** | **(mm)** |  |  |
| Frances | Barley | 0.5 | 50 | 1.0 | 100 | 3.0 | 300 | $110.00 | SA River Murray |
|  | Canola | 0.5 | 50 | 1.5 | 150 | 3.5 | 350 |  |  |
|  | Chickpea | 0.5 | 50 | 1.5 | 150 | 3.0 | 300 |  |  |
|  | Maize | 3.0 | 300 | 6.0 | 600 | 7.0 | 700 |  |  |
|  | Wheat | 0.5 | 50 | 1.5 | 150 | 3.5 | 350 |  |  |
| Kerang | Barley | 0.5 | 50 | 2.0 | 200 | 5.0 | 500 | $95.00 | VIC Murray (Below Choke) |
|  | Canola | 1.0 | 100 | 3.0 | 300 | 6.0 | 600 |  |  |
|  | Chickpea | 0.5 | 50 | 2.0 | 200 | 5.0 | 500 |  |  |
|  | Maize | 3.0 | 300 | 6.5 | 650 | 8.5 | 850 |  |  |
|  | Wheat | 0.5 | 50 | 2.5 | 250 | 5.5 | 550 |  |  |
| Griffith | Barley | 0.5 | 50 | 2.5 | 250 | 5.5 | 550 | $80.00 | NSW Lower Lachlan |
|  | Canola | 1.5 | 150 | 4.5 | 450 | 7.5 | 750 |  |  |
|  | Chickpea | 1.0 | 100 | 3.0 | 300 | 6.0 | 600 |  |  |
|  | Maize | 4.0 | 400 | 8.0 | 800 | 10.0 | 1000 |  |  |
|  | Wheat | 1.0 | 100 | 3.5 | 350 | 6.5 | 650 |  |  |
| Hagley | Barley | 0.5 | 50 | 2.0 | 200 | 4.0 | 400 | $65.00 | TAS North Esk |
|  | Canola | 1.5 | 150 | 3.0 | 300 | 5.5 | 550 |  |  |
|  | Chickpea | 1.0 | 100 | 2.0 | 200 | 4.0 | 400 |  |  |
|  | Maize | 2.5 | 250 | 5.5 | 550 | 8.0 | 800 |  |  |
|  | Wheat | 1.0 | 100 | 2.5 | 250 | 5.0 | 500 |  |  |

**Table S8:** The 16 genotype x management options selected based on crop type, genotype, and watering regime for each region.

| **Crop** | **Genotype** | **Regime** |
| --- | --- | --- |
| Barley | Early | Rainfed |
|  | Late |  |
| Canola | Early | Rainfed |
|  |  | Irrigated |
|  | Late | Rainfed |
|  |  | Irrigated |
| Chickpea | Early | Rainfed |
|  |  | Irrigated |
|  | Late | Rainfed |
|  |  | Irrigated |
| Durum wheat | Early | Rainfed |
|  |  | Irrigated |
|  | Late | Rainfed |
|  |  | Irrigated |
| Maize | Early | Irrigated |
|  | Late |  |

**Table S9:** Long-term seasonal variation in rainfall (mm) across a range of representative environments in Australian irrigated cropping regions under historical (1985-2004) and future (2070-2089) climates.

| Region | Period | Season | Dry | Moderate | Wet |
| --- | --- | --- | --- | --- | --- |
| Hagley TAS | Historical | Winter | 345 | 455 | 580 |
|  |  | Summer | 155 | 235 | 310 |
| Frances SA | Historical | Winter | 300 | 415 | 510 |
|  |  | Summer | 90 | 150 | 220 |
| Griffith NSW | Historical | Winter | 115 | 205 | 320 |
|  |  | Summer | 80 | 145 | 245 |
| Kerang VIC | Historical | Winter | 130 | 205 | 280 |
|  |  | Summer | 70 | 110 | 240 |
| Hagley TAS | Future | Winter | 110 | 185 | 365 |
|  |  | Summer | 70 | 115 | 225 |
| Frances SA | Future | Winter | 110 | 180 | 355 |
|  |  | Summer | 45 | 75 | 150 |
| Griffith NSW | Future | Winter | 55 | 95 | 185 |
|  |  | Summer | 50 | 80 | 160 |
| Kerang VIC | Future | Winter | 60 | 100 | 200 |
|  |  | Summer | 40 | 70 | 135 |

Table S10: Temperature thresholds for frost and heat stress during sensitive Zadoks growth stages with corresponding yield penalties (after Bell *et al.* (2015)).

| Temperature | Stress level | Zadoks sensitive stage | Daily yield penalty (%) |
| --- | --- | --- | --- |
| −2 °C to 0 °C | Mild | 60–69 | 10 |
| −4 °C to −2 °C | Moderate | 60–75 | 20 |
| ≤−4 ℃ | Severe | 60–79 | 90 |
| 32 °C to 34 °C | Mild | 60–79 | 10 |
| 34 °C to 36 °C | Moderate | 60–79 | 20 |
| >36 °C | Severe | 60–79 | 30 |
